# Comparative efficacy and safety of four immunomodulatory agents for acute spinal cord injury: a systematic review and Bayesian network meta-analysis

**DOI:** 10.3389/fphar.2026.1942918

**Published:** 2026-10-01

**Authors:** Yue Peng, Binghan Cai, Yuerong Ren, Qian Jiang, Bing Jiang, Lili Xie

**Affiliations:** 1 Department of Ophthalmology, The Second Xiangya Hospital, Central South University, Changsha, China; 2 Hunan Clinical Research Center of Ophthalmic Disease, Changsha, China

**Keywords:** acute spinal cord injury, Bayesian network meta-analysis, erythropoietin, granulocyte colony-stimulating factor, methylprednisolone, minocycline, neurological recovery

## Abstract

**Study Design:**

Systematic review and Bayesian network meta-analysis.

**Objective:**

To compare the efficacy and safety of methylprednisolone (MP), erythropoietin (EPO), granulocyte colony-stimulating factor (G-CSF), and minocycline for the treatment of acute spinal cord injury (ASCI).

**Methods:**

MEDLINE, Embase, the Cochrane Library, and ClinicalTrials.gov were searched from inception to 15 June 2025. Randomized and nonrandomized comparative studies were eligible. Bayesian random-effects network meta-analyses compared MP, EPO, EPO plus MP, G-CSF, and minocycline. Treatment effects were reported as odds ratios (ORs) or mean differences (MDs) with 95% credible intervals (CrIs). Model convergence and fit, heterogeneity, predictive intervals, and global and local inconsistency were evaluated. Follow-up-stratified and exploratory restriction analyses were performed where feasible. Formal comparison-level Confidence in Network Meta-Analysis (CINeMA) assessment was not feasible; certainty was appraised qualitatively across its six domains.

**Results:**

Twenty comparative reports were included: nine randomized and 11 nonrandomized reports. For neurological grade improvement, G-CSF was associated with higher odds of improvement than the common control node (OR = 5.12, 95% CrI 1.15–32.42). Estimates for EPO (OR = 4.87, 95% CrI 0.44–135.90), EPO plus MP (OR = 3.44, 95% CrI 0.09–164.02), and MP (OR = 1.31, 95% CrI 0.30–5.72) were imprecise and included the null. For change in ASIA Motor Score, G-CSF had the largest point estimate (MD = 11.49, 95% CrI −0.44 to 23.49), followed by minocycline (MD = 6.83, −15.24–28.75), EPO (MD = 6.75, −13.16–26.42), and MP (MD = 2.28, −6.24–10.94); all credible intervals crossed zero. For overall adverse events, the estimate for MP was imprecise (OR = 1.13, 95% CrI 0.58–2.34), while the G-CSF effect was not reliably estimable because near-complete event occurrence produced a highly prior-sensitive posterior. Other safety estimates were imprecise.

**Conclusion:**

G-CSF showed a potential beneficial signal for neurological grade improvement. However, the limited evidence base, inclusion of nonrandomized and nonconcurrent comparisons, substantial heterogeneity, wide credible and predictive intervals, and uncertain transitivity preclude definitive conclusions about comparative efficacy or safety. Treatment rankings should be regarded as exploratory and do not establish the superiority of any intervention.

**Systematic Review registration:**

https://www.crd.york.ac.uk/PROSPERO/view/, identifier CRD420251027856.

## Introduction

Acute spinal cord injury (SCI) can cause persistent motor, sensory, and autonomic impairment below the level of injury, with substantial consequences for patients, families, and health systems (Safdarian et al., 2023; Lu et al., 2025). Primary mechanical damage is followed by a secondary injury cascade involving ischemia, excitotoxicity, calcium dysregulation, oxidative stress, and inflammation (Ahuja et al., 2017; Hu et al., 2023).

Inflammation after SCI is temporally and phenotypically heterogeneous. Early neutrophil and monocyte infiltration can amplify barrier disruption, edema, and neural cell loss through cytokine and oxidative pathways, whereas immune and glial responses also contribute to debris clearance, remodeling, and repair later in the response (Ahuja et al., 2017; Hellenbrand et al., 2021; Sofroniew, 2018). The familiar M1/M2 shorthand does not capture the full continuum of myeloid states. A plausible therapeutic objective is therefore context- and time-dependent modulation rather than indiscriminate suppression.

Several agents with immunomodulatory or neuroprotective actions have been evaluated in acute SCI. High-dose MP was widely adopted after the NASCIS trials, but its efficacy and safety remain controversial (Donovan and Kirshblum, 2018; Bracken, 2012; Liu et al., 2019). EPO, G-CSF, and minocycline have distinct pharmacological actions that may influence components of secondary injury (Matis and Birbilis, 2009; Kabu et al., 2015; Kawabe et al., 2011; Hu et al., 2025; Wells et al., 2003). Clinical evidence remains limited and heterogeneous, and direct comparisons among these agents are sparse (Kabu et al., 2015; Bowes and Yip, 2014).

We therefore conducted a systematic review and Bayesian network meta-analysis to compare the efficacy and safety of MP, EPO, G-CSF, and minocycline, with EPO plus MP represented as a separate combination node. The objective was to estimate relative treatment effects while presenting the uncertainty and limitations of the available evidence explicitly.

## Methods

The review was registered in PROSPERO (CRD420251027856) and is reported in accordance with the PRISMA extension for network meta-analyses (Hutton et al., 2015). Because only aggregate data from published reports were used, ethics approval and individual informed consent were not required.

### Search strategies and inclusion criteria

We systematically searched MEDLINE, Embase, the Cochrane Library, and ClinicalTrials.gov from database inception through 15 June 2025. We also screened the reference lists of included studies for additional reports. No language restrictions were imposed during the electronic searches. Search strategies for the bibliographic databases are provided in Supplementary Appendix 1.

Studies were eligible if (1) participants had acute SCI; (2) the active regimen was MP, EPO, EPO plus MP, G-CSF, or minocycline; (3) the comparator was placebo, standard care, an untreated or no-steroid group, a historical or sequential-period comparator, or another defined network treatment; (4) at least one eligible efficacy or safety outcome was reported; and (5) the report provided a randomized or nonrandomized comparative analysis. An early-phase single-arm trial report was eligible only when it included an analyzable external comparator relevant to the network. Nonconcurrent comparisons were retained because randomized evidence was limited, but their susceptibility to temporal confounding was considered in the ROBINS-I assessment and interpretation.

Treatment-node and co-intervention handling was defined as follows. MP and methylprednisolone sodium succinate were grouped within the MP node. The common control node incorporated placebo, saline control, standard care, as well as untreated, no-medication, or no-steroid comparators; this analytical label did not indicate that all control groups received placebo. EPO combined with MP was modeled as a separate combination node. Reported background care administered consistently across study arms was treated as a co-intervention. Where reporting on co-intervention was lacking, balanced co-intervention provision across groups was not assumed. Pharmacological agents and regimens beyond the predefined scope of intervention, including riluzole, were excluded from the quantitative synthesis.

### Study selection and data extraction

Records were imported into EndNote X9 and deduplicated. Two reviewers independently screened titles and abstracts, assessed potentially eligible full texts, and determined inclusion. Disagreements were resolved by discussion or, when necessary, by a third reviewer.

Two reviewers independently extracted data using a piloted standardized data extraction form. Extracted information comprised study identifiers and setting; baseline neurological severity and neurological level, where reported; injury mechanism; intervention timing; surgical management; mean arterial pressure management; randomized, enrolled, and analyzed denominators; analysis population; intervention and comparator definitions; whether comparators were concurrent, historical, or sequential; blinding; follow-up time points; and outcome definitions. Neurological outcomes included AIS or Frankel grade change, alongside ASIA or NASCIS motor and sensory scores, recorded according to the terminology adopted in each primary report. Safety outcomes encompassed mortality, adverse events, infections, gastrointestinal complications, and thromboembolic events.

### Assessment of bias

Two reviewers independently assessed risk of bias. Randomized trials were evaluated with RoB 2 (Sterne et al., 2019), covering randomization, deviations from intended interventions, missing outcome data, outcome measurement, and selection of reported results. Domain and overall judgments were categorized as low risk, some concerns, or high risk. Nonrandomized studies were evaluated with ROBINS-I (Sterne et al., 2016), including bias due to confounding, participant selection, intervention classification, deviations from intended interventions, missing data, outcome measurement, and selective reporting. Disagreements were resolved by consensus or third-reviewer adjudication. Study-level risk-of-bias assessments are provided in Supplementary Appendix 2.

### Outcomes

The coprimary efficacy outcomes were improvement by at least one grade on the neurological impairment scale reported by the study and change from baseline in ASIA Motor Score at an eligible follow-up. AIS and Frankel responses, unthresholded grade change, and NASCIS scores were recorded separately at extraction and were not treated as interchangeable without an explicit harmonization rule. Secondary efficacy outcomes included sensory scores and activities of daily living measured with the Barthel Index or Functional Independence Measure. Safety outcomes included mortality, overall adverse events, infection, gastrointestinal bleeding, and thromboembolic events. Study-specific outcome terminology is retained in Table 1.

**TABLE 1:** Characteristics of studies included in the meta-analysis.

Study	Country	Study design	Comparison (sample size)	Follow-up (time points)	Reported outcomes
Mounissamy et al. (2025)	India	Single-center, single-blind, randomized, placebo-controlled trial	G-CSF (n = 16) vs. normal saline placebo (n = 18)	1, 3, and 6 months	AIS grade change
Inada et al. (2014)	Japan	Multicenter, prospective, open-label, nonrandomized controlled clinical trial	G-CSF (n = 17) vs. no-G-CSF control (n = 24)	1 week; 3, 6, and 12 months	ASIA motor score; AIS grade improvement by ≥ 1 grade
Kamiya et al. (2015)	Japan	Retrospective comparative study with a historical MPSS control cohort	G-CSF (n = 28) vs. historical MPSS cohort (n = 34)	3 months	ASIA motor score; AIS grade improvement
Koda et al. (2021)	Japan	Prospective, multicenter, randomized, double-blind, placebo-controlled, parallel-group phase III trial	G-CSF (FAS, n = 43) vs. placebo (FAS, n = 45)	3, 6, and 12 months	ASIA motor score; AIS grade improvement by ≥ 1 grade (3 and 6 months); any adverse event; infection
Takahashi et al. (2012)	Japan	Open-label, single-arm, dose-escalation phase I/IIa trial with an exploratory historical MPSS comparison	G-CSF 10 μg/kg subgroup (n = 11) vs. historical MPSS cohort (n = 28); total G-CSF trial population, n = 16	Up to 3 months	ASIA motor score; AIS grade improvement by ≥ 1 grade
Dalla Costa et al. (2015)	Italy	Multicenter, randomized, outcome assessor–blinded, parallel-group, active-controlled trial	EPO (n = 11) vs. MP (n = 8)	Days 3, 7, and 14; months 1, 2, and 3	ASIA motor score; AIS grade improvement by ≥ 1 grade
Ganjeifar et al. (2021)	Iran	Single-center, pilot, double-blind, randomized, parallel-group add-on controlled trial	EPO + MP (n = 27) vs. MP alone (n = 27)	6 and 12 months	AIS grade–based neurological outcome; sphincter function; 1-year mortality
Alibai et al. (2014)	Iran	Two-center, prospective, randomized, double-blind, placebo-controlled pilot trial	EPO + MPSS (n = 15) vs. saline placebo + MPSS (n = 15)	1 week; 1 and 6 months	AIS grade improvement by ≥ 1 grade
Cheng et al. (2016)	China	Retrospective comparative cohort study with a saline-treated control group	EPO + standard care (n = 30) vs. saline control + standard care (no EPO; n = 30)	12 and 24 months	AIS grade improvement by ≥ 1 grade; ASIA motor and sensory scores
Ito et al. (2009)	Japan	Single-center, prospective, nonrandomized consecutive cohort study comparing sequential treatment periods	MPSS (n = 38) vs. no-MPSS control (n = 41)	3 months	ASIA motor score; AIS grade change; infection categories
Suberviola et al. (2008)	Spain	Single-center retrospective ICU cohort study with an untreated comparator	MP (n = 59) vs. no-MP control (n = 23)	At ICU discharge (<1 month)	Frankel grade improvement by ≥ 1 grade; respiratory and total infections
Matsumoto et al. (2001)	Japan	Prospective, randomized, double-blind, placebo-controlled trial	MPSS (n = 23) vs. placebo (n = 23)	Up to 2 months	Complications/adverse events
Tsutsumi et al. (2006)	Japan	Single-center retrospective comparative cohort study	MPSS (n = 37) vs. no-MPSS control (n = 33)	6 weeks and 6 months	ASIA motor score; early complications
Evaniew et al. (2015)	Canada	Multicenter propensity score–matched cohort study using prospectively collected registry data	MPSS (n = 44) vs. no-steroid control (n = 44)	Neurological: Median 127 vs. 117 days; safety: during hospitalization	ASIA motor score; total in-hospital complications
Bracken et al. (1992)	United States	One-year follow-up of the multicenter, randomized, double-blind, three-arm, placebo-controlled NASCIS II trial	MPSS (randomized, n = 162) vs. placebo (randomized, n = 171)	6 weeks; 6 and 12 months	NASCIS motor and sensory scores; mortality and morbidity
Sunshine et al. (2017)	United States	Single-center retrospective registry-based cohort study with concurrent MPSS-exposed and unexposed groups	MPSS-exposed (n = 160) vs. unexposed (n = 151)	At rehabilitation discharge (months not reported)	ASIA motor score; infections; gastrointestinal complications
Pointillart et al. (2000)	France	Prospective, randomized, four-arm, parallel-group controlled trial with blinded outcome assessment	MPSS (n = 27) vs. no-medication control (n = 25)	12 months	ASIA motor score
Gerhart et al. (1995)	United States	Population-based retrospective cohort study using statewide SCI surveillance and medical-record data	MPSS (n = 188) vs. no-MPSS control (n = 90); neurologically evaluable, n = 175 vs. 76	At hospital discharge (months not reported)	Frankel grade improvement by ≥ 1 grade
Gerndt et al. (1997)	United States	Retrospective review with a historical control group	MPSS (n = 93) vs. historical no-steroid control (n = 47)	During hospitalization and rehabilitation (months not reported)	Pneumonia; durations of ventilation, ICU stay, and rehabilitation; mortality
Casha et al. (2012)	Canada	Single-center, randomized, double-blind, placebo-controlled phase II trial	Minocycline (n = 27) vs. saline placebo (n = 25)	12 months	ASIA motor score; safety/adverse events

Abbreviations: AIS, American Spinal Injury Association Impairment Scale; ASIA, American Spinal Injury Association; EPO, erythropoietin; FAS, full analysis set; G-CSF, granulocyte colony-stimulating factor; ICU, intensive care unit; MP, methylprednisolone; MPSS, methylprednisolone sodium succinate; NASCIS, National Acute Spinal Cord Injury Study. Comparator labels reflect the original reports; untreated, no-steroid, and no-medication controls were not placebo controls. Reported outcomes are summarized at the study level; listing indicates that an outcome was reported, not that the intervention had a favorable effect. For Takahashi et al., n = 11 is the 10 μg/kg 10 μg/kg G-CSF subgroup used in the exploratory historical comparison; the phase I/IIa trial enrolled 16 participants. For Bracken et al., the naloxone arm (n = 154) is not shown. For Pointillart et al., the nimodipine and MPSS + nimodipine arms (n = 27 each) are not shown because they were outside the prespecified network domain.

### Data synthesis and analysis

All Bayesian analyses and diagnostic cross-checks were performed in R version 4.4.3 using gemtc 1.1-0, JAGS 4.3.2, and netmeta 3.2-0. Continuous outcomes were summarized as mean differences (MDs), while dichotomous efficacy and safety outcomes were expressed as odds ratios (ORs). Bayesian network estimates were reported with 95% credible intervals (CrIs).

Bayesian hierarchical random-effects models were used to synthesize direct and indirect evidence across MP, EPO, EPO plus MP, G-CSF, minocycline, and the unified control node. Binomial likelihoods with logit links were specified for dichotomous outcomes, and normal likelihoods with identity links for continuous outcomes. Four Markov chains ran for 50,000 adaptation iterations, followed by 150,000 monitored sampling iterations per chain; every 10th draw was retained, yielding 15,000 draws per chain and 60,000 samples per fitted model. A fixed random seed of 20260807 was applied. Heterogeneity-prior sensitivity analyses employed the alternative prior distributions detailed in Supplementary Appendix 6, Supplementary Table S4.

Convergence was assessed by trace plots and formally using three prespecified criteria applied to every monitored parameter: the upper 97.5% bound of the Gelman–Rubin–Brooks potential scale reduction factor (PSRF) below 1.05, effective sample size (ESS) above 1,000, and Monte Carlo standard error (MCSE) below 5% of the posterior standard deviation. Model fit was evaluated using residual deviance and the deviance information criterion (DIC) for fixed-effect, random-effects, and, where applicable, unrelated mean effects (UME) models. Between-study heterogeneity was characterized via the posterior distribution of τ, accompanied by predictive intervals and prior sensitivity analyses (Deeks et al., 2024). Global inconsistency was evaluated by consistency-versus-UME DIC comparison in loop-containing networks. Local inconsistency was assessed by node splitting; the reported statistic corresponds to the two-sided posterior tail-area probability 2 × min{P(IF > 0), P(IF < 0)}, where IF denotes the inconsistency factor. Frequentist design-by-treatment interaction and net-splitting analyses were used as descriptive cross-checks. Importantly, absence of statistical significance was not interpreted as proof of consistency.

Follow-up-stratified analyses were only undertaken when the network remained connected. Formal evaluation of transitivity was not feasible, given inconsistent reporting of baseline AIS grade, neurological level, injury mechanism, surgical management, intervention timing, mean arterial pressure protocols, and outcome-assessment methods; strata defined by pairwise comparisons were too sparse to support reliable network meta-regression. Transitivity was therefore treated as uncertain. Within each analytical framework, a single follow-up timepoint was selected per study; separate short-term and long-term network analyses were conducted where possible. Exploratory restriction analyses are presented in Supplementary Appendix 7.

Network effect estimates were presented in forest plots and league tables. Surface under the cumulative ranking curve (SUCRA) values summarized posterior rankings, though rankings were considered secondary and exploratory. A formal CINeMA assessment was not conducted, owing to the unavailability of comparison-specific contribution matrices, contribution-weighted study-level risk-of-bias judgments, and predefined clinical equivalence margins. A structured qualitative appraisal organized around the six CINeMA domains is presented in Supplementary Appendix 8; this appraisal does not constitute a formal CINeMA grade (Nikolakopoulou et al., 2020).

Exploratory assessments of small-study effects and reporting bias are summarized in Supplementary Appendix 3. Full diagnostic output—including network geometry, parameter-level Markov chain Monte Carlo convergence, model fit, heterogeneity and prior sensitivity, predictive intervals, global and local inconsistency, frequentist cross-checks, and the input data audit records—is provided in Supplementary Appendix 6 (Supplementary Tables S1–S10 and accompanying detail sheets).

## Results

### Search results and study characteristics

The study-selection process is summarized in Figure 1. The search identified 1,116 records; 881 remained after duplicate removal, 79 full texts were assessed, and 20 reports met the eligibility criteria. The evidence base comprised nine randomized trials (Pointillart et al., 2000; Bracken et al., 1992; Matsumoto et al., 2001; Mounissamy et al., 2025; Koda et al., 2021; Dalla Costa et al., 2015; Alibai et al., 2014; Ganjeifar et al., 2021; Casha et al., 2012) and 11 nonrandomized reports (Sunshine et al., 2017; Evaniew et al., 2015; Tsutsumi et al., 2006; Suberviola et al., 2008; Ito et al., 2009; Gerndt et al., 1997; Gerhart et al., 1995; Kamiya et al., 2015; Inada et al., 2014; Takahashi et al., 2012; Cheng et al., 2016). These reports evaluated MP, EPO, G-CSF, and minocycline, with EPO plus MP represented as a separate active node. A de-duplicated participant total is not reported because the source reports used different denominators and analysis populations, and overlap among linked Japanese G-CSF reports could not be excluded from the publications alone. Study characteristics and study-specific denominators are presented in Table 1.

**FIGURE 1:**
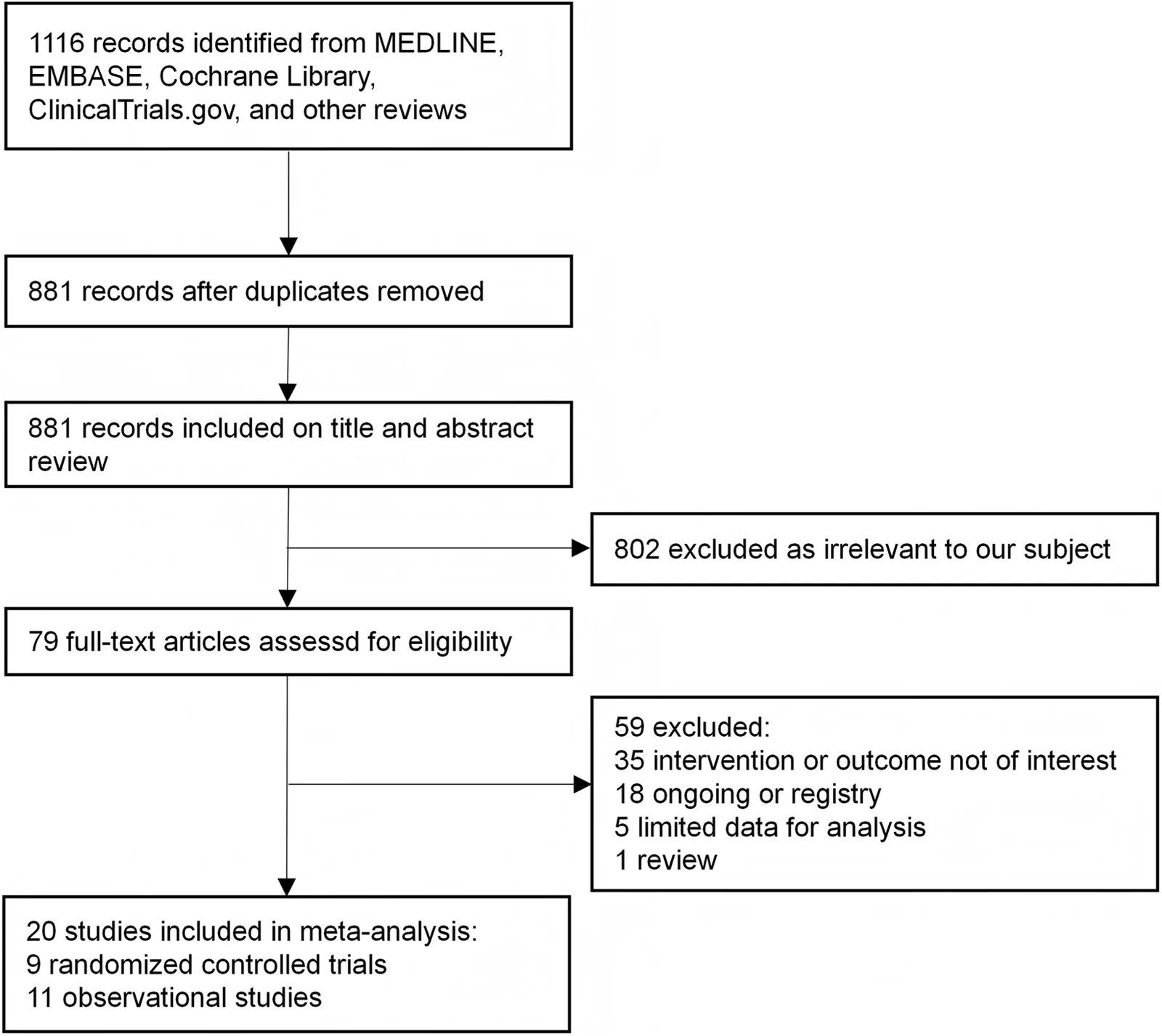
PRISMA flow diagram of study identification, screening, eligibility assessment, and final inclusion. The flow structure follows the PRISMA extension guidelines for network meta-analyses (Hutton et al., 2015). Figure generated independently by the authors based on the review’s literature search and screening records; no external published material was reproduced or adapted.

### Eligibility assessment of riluzole and combination regimens

RISCIS met the population, comparator, outcome, and design domains but was not entered into the network because riluzole lay outside the intervention domain applied in this review. EPO plus MP was retained as a separate node and was evaluated in two randomized trials by Alibai et al. (2014) and Ganjeifar et al. (2021). Other drug combinations were outside the defined intervention domain; this review therefore cannot support conclusions about their efficacy.

### Risk of bias assessment

Risk of bias was evaluated with RoB 2 for randomized trials and ROBINS-I for nonrandomized studies. Of the nine randomized trials, eight were judged to raise some concerns and one was judged to be at high risk of bias. Common limitations were incomplete reporting of randomization and allocation concealment, lack of blinding, and unclear handling of missing outcome data.

The 11 nonrandomized studies had higher risks of bias: two were judged to have moderate risk and nine serious risk. The principal concerns were confounding, selection of participants, nonrandom treatment allocation, historical or sequential-period comparisons, baseline imbalance, and limited statistical adjustment. No nonrandomized study was judged to have low risk of bias.

### Model diagnostics, heterogeneity, inconsistency, and transitivity

All 12 primary random-effects models satisfied the PSRF threshold; ten fulfilled all three prespecified criteria. The short-term AIS model and overall adverse events model yielded ESS values of 766 and 966, respectively, with MCSEs equivalent to 3.6% and 3.2% of the corresponding posterior standard deviations. In total, 62 out of 71 fitted models met all three convergence criteria. Residual deviance per data point for primary models ranged from 0.775 to 1.129. Four of 12 networks formed closed loops. In these networks, consistency and UME models exhibited comparable goodness-of-fit, with absolute DIC differences between 0.86 and 2.15. Nevertheless, the two AIS UME models did not meet the ESS criterion. Node-splitting analysis was performed for 20 contrasts amenable to splitting; six contrasts were not interpreted owing to weak identification of direct or dependent indirect evidence. For the remaining 14 contrasts, the 95% CrI for each inconsistency factor spanned zero, and two-sided posterior tail-area probabilities ranged from 0.2317 to 0.9869. Between-design interaction p-values ranged from 0.3010 to 0.8788. These analyses revealed no robust evidence of inconsistency, though a null finding cannot confirm consistency (Supplementary Appendix 4). Marked between-study heterogeneity was observed in the overall AIS and motor networks (τ = 1.11 and 8.65 points, respectively) (Supplementary Appendix 5). Transitivity remained uncertain, as critical effect modifiers were inconsistently reported and comparison-specific strata were too sparse to enable reliable evaluation (Supplementary Appendices 6, 7).

### Neurological grade improvement by at least one grade

Figure 2A presents the treatment network for neurological grade improvement. Relative to the unified control node (placebo or standard care), G-CSF was associated with higher odds of improvement (OR = 5.12, 95% CrI 1.15–32.42) (Figure 3A). Effect estimates for EPO (OR = 4.87, 95% CrI 0.44–135.90), EPO plus MP (OR = 3.44, 95% CrI 0.09–164.02), and MP (OR = 1.31, 95% CrI 0.30–5.72) were highly imprecise and spanned the null value (Figure 4A). Although the effect interval for G-CSF excluded the null, the interval remained wide; potential overlapping study cohorts across linked publications further constrain the interpretability of the observed precision.

**FIGURE 2:**
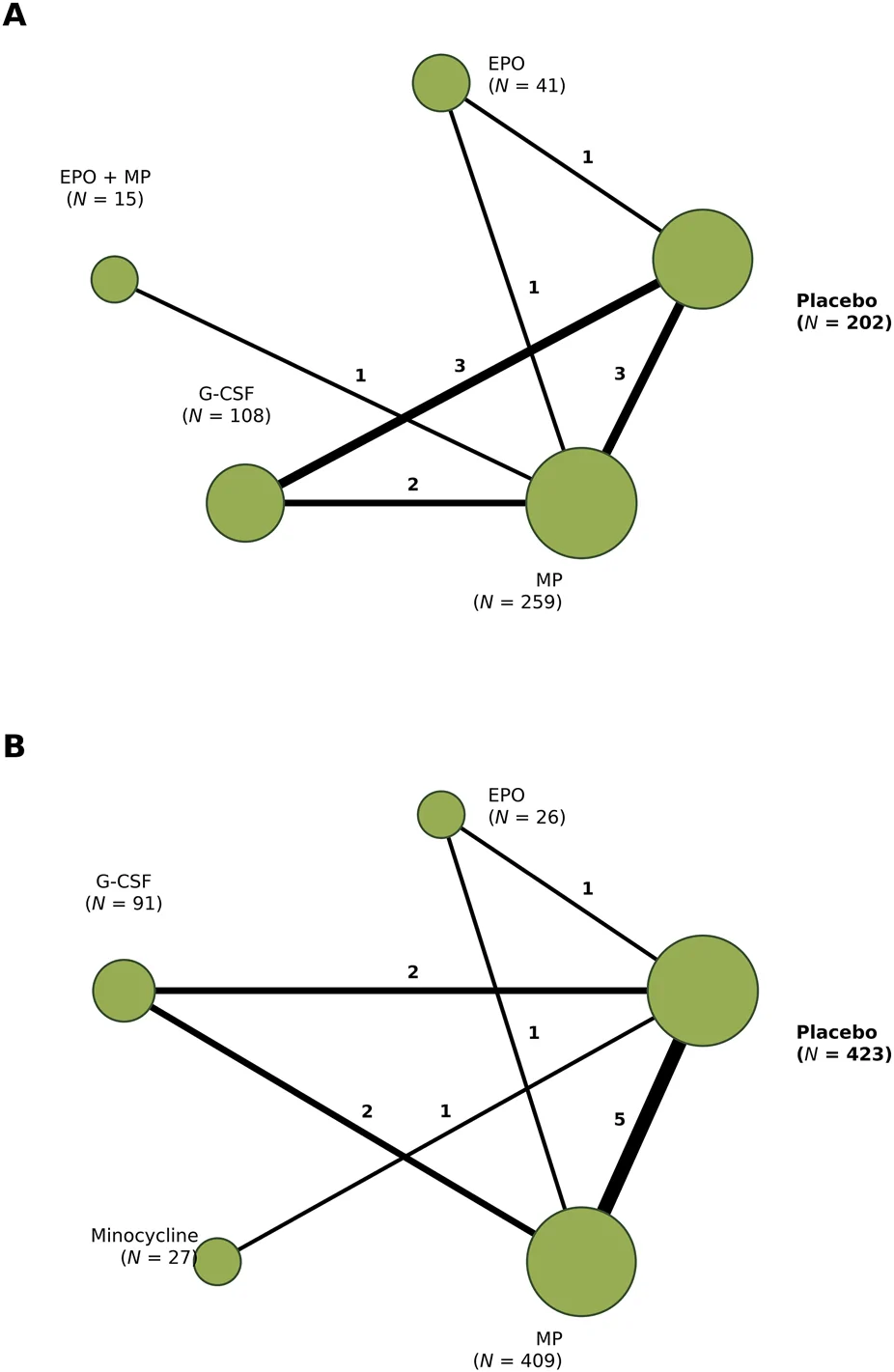
Network plots for efficacy outcomes. **(A)** Neurological grade improvement of at least one grade. **(B)** Mean change in ASIA Motor Score. Node size is proportional to the sample size encoded in the outcome-specific analysis dataset; edge width is proportional to the number of contributing studies, which is printed beside each edge. Counts are not de-duplicated across potentially linked reports. Source: author-generated in R/Python from data extracted from the included reports listed in Table 1. No external figure was reproduced or adapted.

**FIGURE 3:**
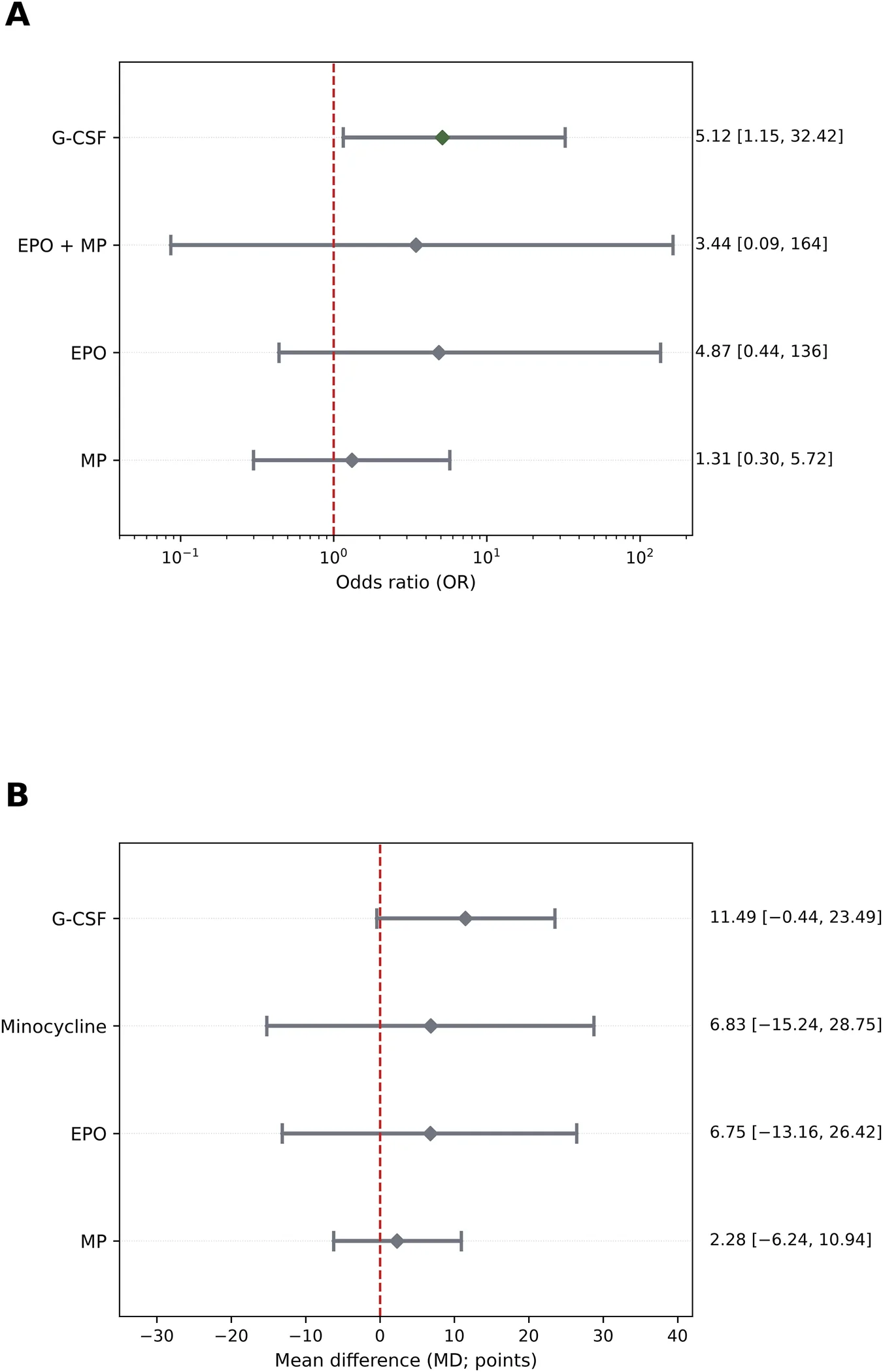
Forest plots of random-effects network estimates for efficacy outcomes: **(A)** neurological grade improvement of at least one grade (odds ratio); **(B)** mean change in ASIA Motor Score (mean difference, points). Active interventions are relative to the common control node. Source: author-generated from the posterior draws of the primary analyses using data extracted from the included reports listed in Table 1. No external figure was reproduced or adapted.

**FIGURE 4:**
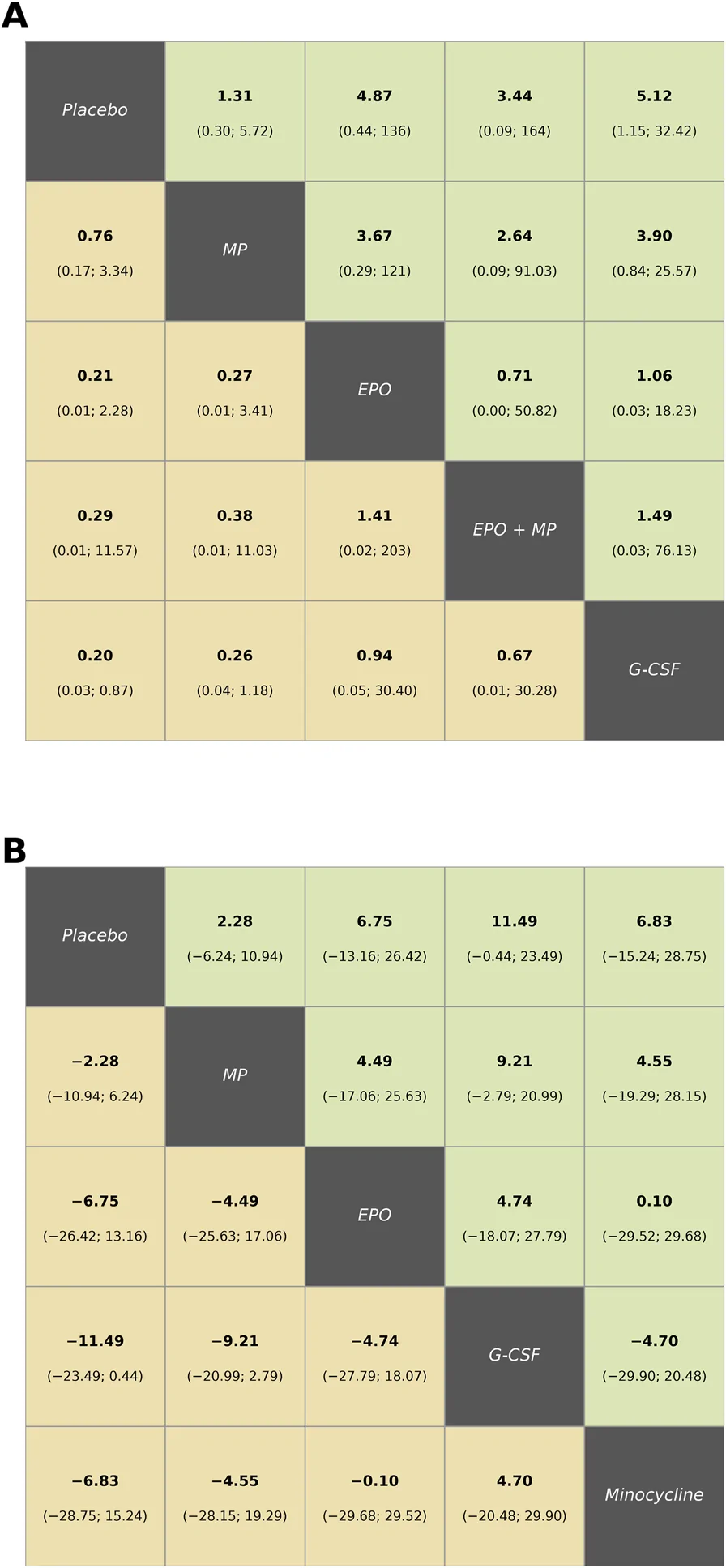
League tables for efficacy outcomes: **(A)** Neurological grade improvement of at least one grade; **(B)** Mean change in ASIA Motor Score. Each off-diagonal cell reports the column treatment relative to the row treatment; opposite triangles are reciprocal for odds ratios and sign-reversed for mean differences. Source: author-generated from 60,000 retained posterior draws per primary model using data extracted from the included reports listed in Table 1. No external figure was reproduced or adapted.

### Mean change in ASIA Motor Score

Figure 2B shows the network for mean change in ASIA Motor Score. Relative to the unified control node, G-CSF yielded the largest point estimate (MD = 11.49, 95% CrI −0.44 to 23.49), followed by minocycline (MD = 6.83, 95% CrI −15.24 to 28.75), EPO (MD = 6.75, 95% CrI −13.16 to 26.42), and MP (MD = 2.28, 95% CrI −6.24 to 10.94) (Figure 3B). All 95% CrIs crossed zero. No active-versus-active pairwise comparison in the league table demonstrated a statistically credible difference between interventions (Figure 4B).

### Safety

Safety outcome networks are shown in Figure 5. Studies with zero events across all treatment arms provide no statistical information for conventional relative OR calculations and were therefore excluded from the corresponding pairwise contrasts. For overall adverse events, the pooled effect estimate for MP was imprecise (OR = 1.13, 95% CrI 0.58–2.34). The comparative safety profile of G-CSF could not be reliably estimated: the only informative study reported adverse events in all 43 patients receiving G-CSF and 44 out of 45 controls, generating an extremely broad, prior-sensitive posterior distribution (Figure 6A). For infectious complications, effect estimates remained imprecise for MP (OR = 1.73, 95% CrI 0.69–4.77), G-CSF (OR = 0.93, 95% CrI 0.11–7.65), and minocycline (OR = 0.38, 95% CrI 0.04–3.45) (Figure 6B). Collectively, the available data failed to identify credible differences in safety profiles across the evaluated interventions; comparative league tables are presented in Figure 7.

**FIGURE 5:**
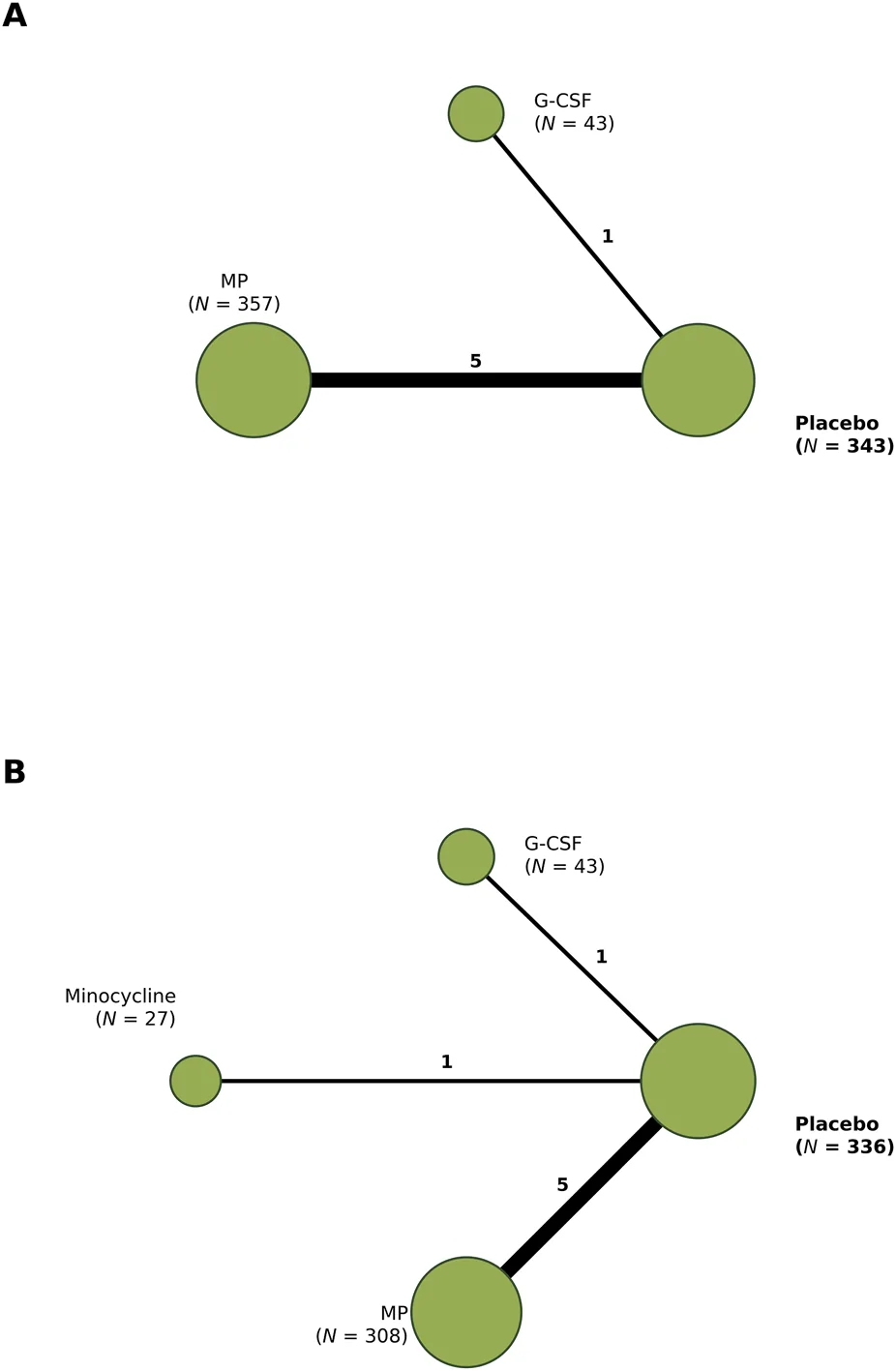
Network plots for safety outcomes. **(A)** Overall adverse events. **(B)** Infection. Node size is proportional to the sample size encoded in the outcome-specific analysis dataset; edge width is proportional to the number of contributing studies, which is printed beside each edge. Source: author-generated in R/Python from data extracted from the included reports listed in Table 1. No external figure was reproduced or adapted.

**FIGURE 6:**
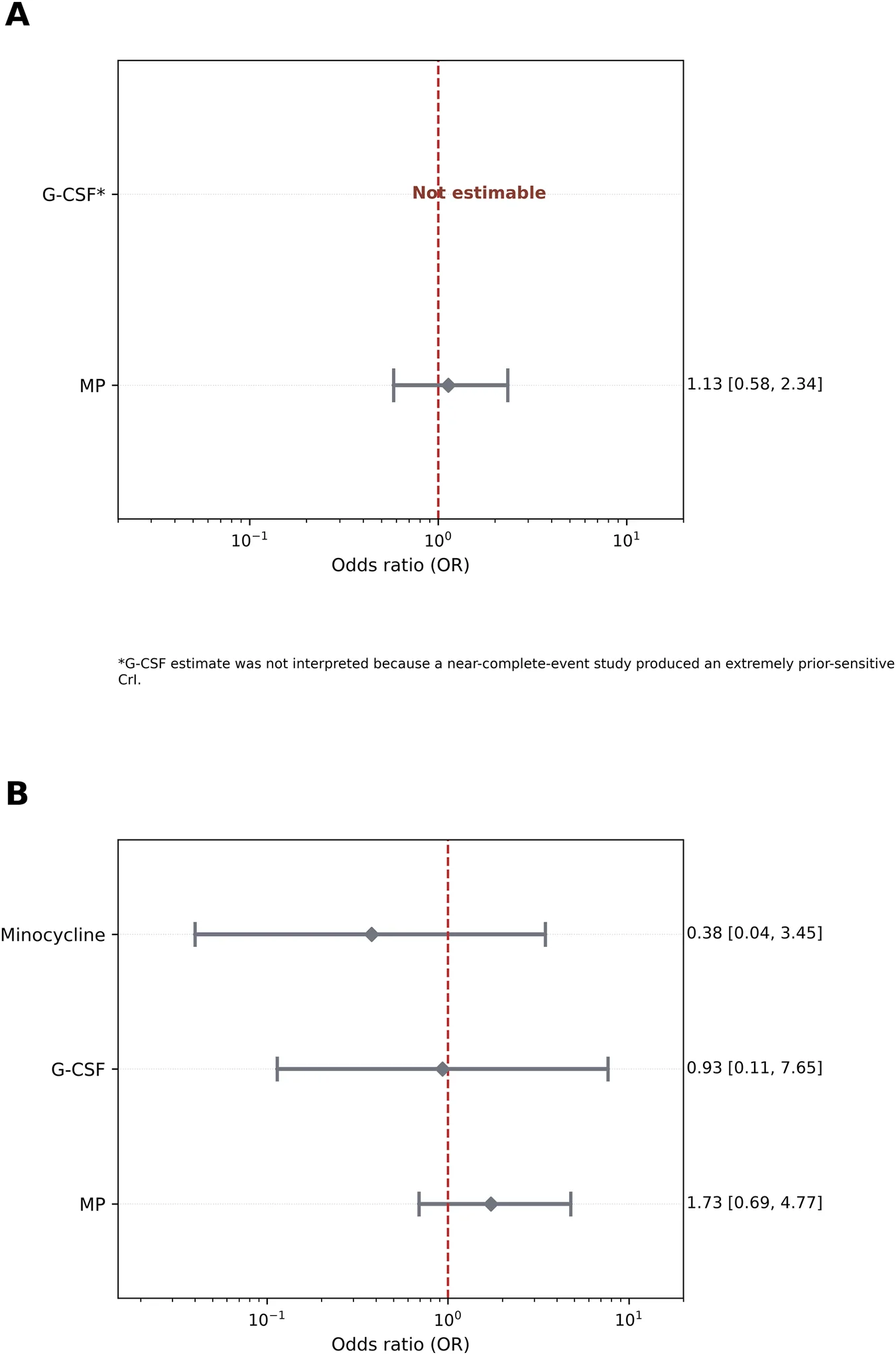
Forest plots of random-effects network estimates for safety outcomes: **(A)** overall adverse events; **(B)** infectious complications. The G-CSF overall-adverse-event contrast is labelled non-estimable, as sparse data with near-complete event rates yielded an extremely wide, prior-sensitive posterior. Figure generated from posterior outputs of primary network analyses based on included study data (see Table 1). No external figure was reproduced or adapted.

**FIGURE 7:**
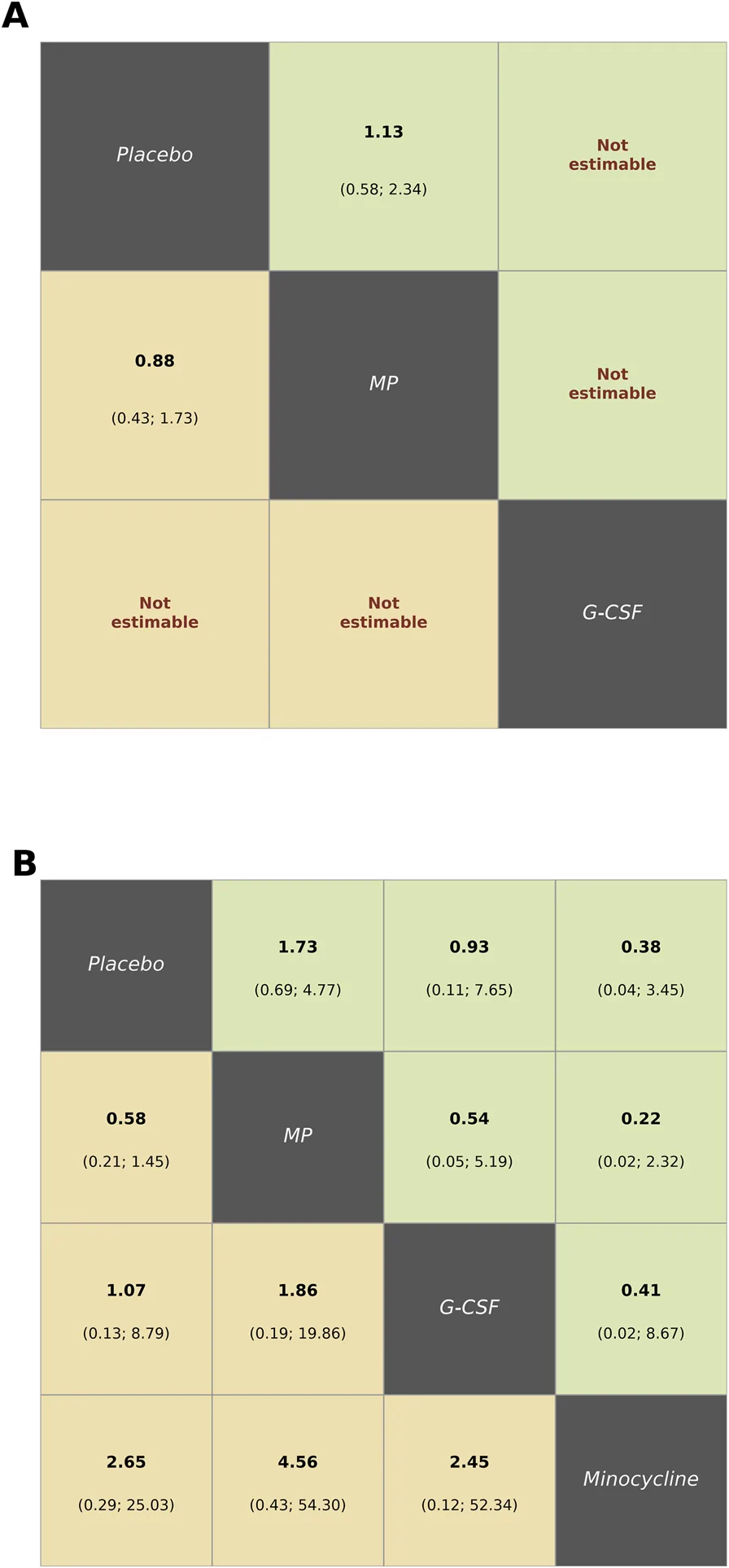
League tables for comparative safety outcomes: **(A)** overall adverse events; **(B)** infectious complications. Each off-diagonal cell reports the column treatment relative to the row treatment. Panel A contains only placebo/standard care, MP, and G-CSF because other agents lacked usable connected data; G-CSF cells are marked not estimable because the posterior was dominated by sparse near-complete-event data. Panel B additionally contains minocycline. Source: author-generated from the posterior draws of the primary analyses using data extracted from the included reports listed in Table 1. No external figure was reproduced or adapted.

### Rank probabilities

SUCRA analyses ranked G-CSF the highest for both neurological grade improvement (0.76) and ASIA Motor Score (0.82). EPO ranked second for neurological grade improvement (SUCRA 0.71; Figure 8A), whereas minocycline had an intermediate rank for ASIA Motor Score (SUCRA 0.57; Figure 8B). For infectious complication outcomes, minocycline ranked highest (0.83), though its corresponding effect estimate was highly imprecise (Figure 8D). The SUCRA ranking of G-CSF for overall adverse events was strongly influenced by prior specifications and thus not clinically interpretable (Figure 8C). All treatment rankings present exploratory summaries of posterior ordering and should be interpreted cautiously given the sparsity of the included networks.

**FIGURE 8:**
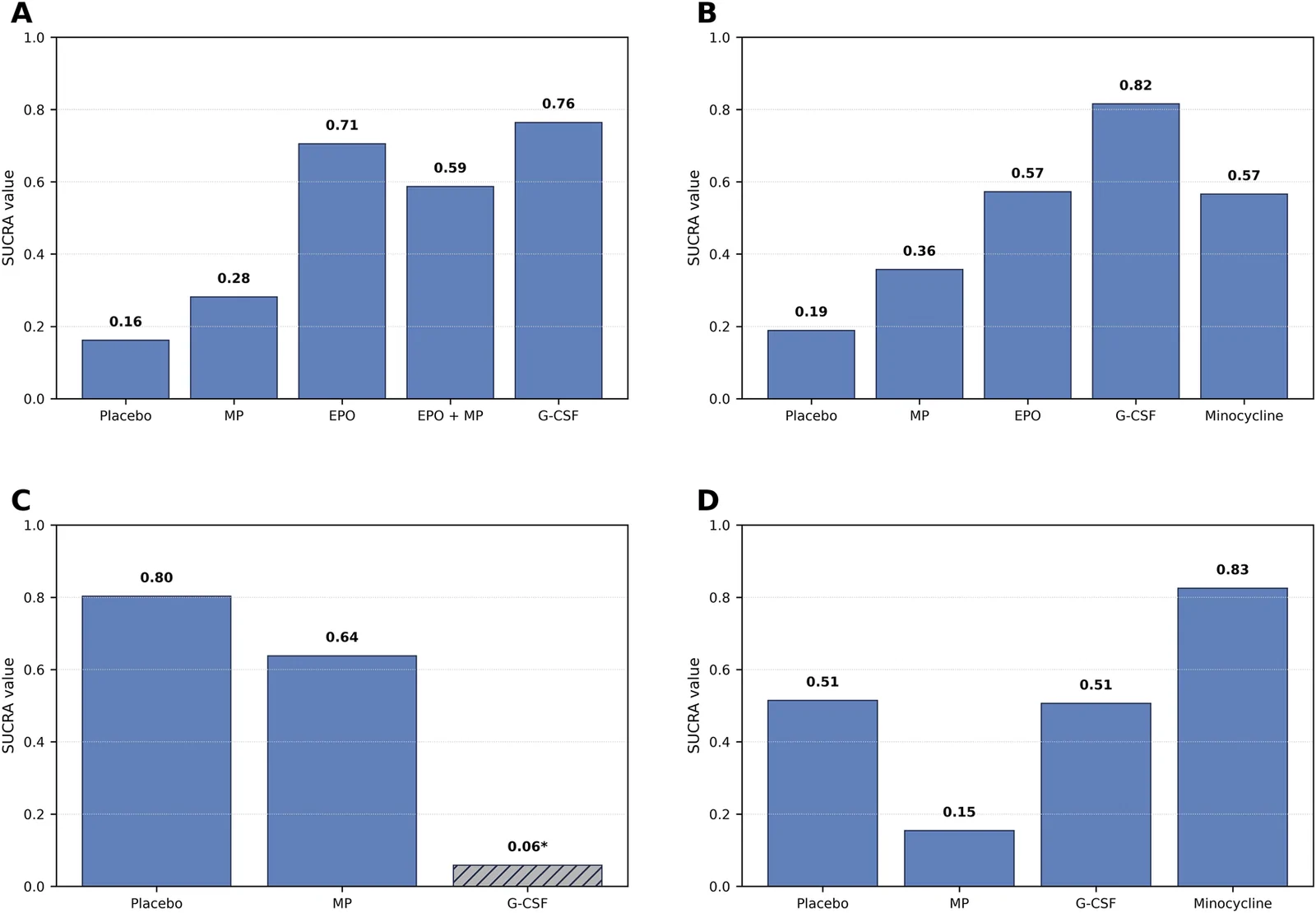
SUCRA rank probability values for evaluated interventions across clinical outcomes. **(A)** Neurological grade improvement. **(B)** ASIA Motor Score. **(C)** Overall adverse events. **(D)** Infectious complications. Higher values indicate a more favorable rank within the fitted network; for safety outcomes, lower event risk is favorable. SUCRA rankings are exploratory only and do not confirm definitive clinical superiority. The hatched bar for G-CSF in the overall adverse-event analysis denotes a highly prior-sensitive, non-interpretable ranking. All SUCRA outputs were generated from primary model posterior draws using data extracted from the included reports listed in Table 1. No external figure was reproduced or adapted.

## Discussion

This network meta-analysis compared five active therapeutic regimens derived from four prespecified agents for ASCI. G-CSF yielded a promising signal for neurological grade improvement relative to the unified control node (OR = 5.12, 95% CrI 1.15–32.42), though the corresponding estimate for ASIA motor score change crossed the null. All remaining active intervention comparisons and interpretable safety contrasts presented highly imprecise effect estimates. These findings are hypothesis-generating rather than definitive enough to establish a robust treatment hierarchy for ASCI neuroprotection.

The present review did not aim to encompass all investigational neuroprotective therapies for ASCI. Instead, its key strength lies in the structured Bayesian comparison of five regimens within a constrained, predefined intervention domain, with comprehensive and transparent quantification of statistical and clinical uncertainty. The available evidence network does not support designation of a superior treatment strategy. Treatment rankings are particularly fragile given the sparse study contribution to several network nodes, substantial between-study heterogeneity, wide credible and predictive intervals, and unresolved uncertainty regarding transitivity across comparisons.

The clinical findings should be interpreted alongside mechanistic uncertainty. Preclinical evidence suggests that G-CSF may protect oligodendrocytes, influence macrophage and microglial responses, mobilize bone-marrow-derived cells, and promote angiogenesis (Kabu et al., 2015; Kawabe et al., 2011; Kadota et al., 2012; Kigerl et al., 2009). However, these mechanisms do not establish mediation of the clinical effects observed in the network, and a recent broad systematic review found heterogeneous and often inconclusive clinical evidence across neuroprotective agents (Serag et al., 2024).

The network did not demonstrate a clear neurological advantage for high-dose MP. Its safety rankings were unfavorable, but the relative estimates were imprecise and do not prove that MP is more harmful than every alternative. The broader literature likewise describes uncertain neurological benefit and concern about complications (Bracken, 2012; Liu et al., 2019). Decisions about MP should therefore not be based on rank probabilities alone; timing, dose, contemporary supportive care, and individual risk–benefit considerations remain important. Mechanistic arguments cannot resolve the uncertainty in the clinical evidence.

EPO and minocycline occupied intermediate positions in the rank distributions, but their wide CrIs do not establish comparative benefit. Minocycline has biological plausibility (Hu et al., 2025), yet the clinical network included only a small phase II trial (Casha et al., 2012). Larger trials with harmonized neurological and safety outcomes are needed before either agent can be recommended on the basis of this network.

Safety outcome interpretation was substantially constrained by sparse event data, inconsistent adverse event definitions across primary studies, and the presence of he presence of double-zero or near-complete-event studies. Sparse-event networks can be particularly sensitive to model choice and prior specification (Evrenoglou et al., 2022). The comparative overall adverse event profile of G-CSF could not be reliably estimated and was deemed unsuitable for clinical interpretation, despite transparent reporting of model-derived ranking outputs. Similarly, infection-related effect estimates were too imprecise to differentiate meaningful therapeutic benefit or harm. Future clinical trials should implement pre-specified, uniform adverse event definitions and comprehensive safety surveillance to mitigate these limitations.

Several limitations materially affect the overall interpretation of the present analyses. First, the evidence base incorporated both randomized and nonrandomized comparative data, including four nonrandomized comparisons relying on historical or sequential-period controls, which increases susceptibility to secular trends, selection bias, and residual confounding. Second, overlapping investigator groups and recruitment periods across included linked Japanese G-CSF reports precluded exclusion of duplicate participant cohorts, which may have artificially inflated precision of the pooled estimates. Third, baseline AIS grade, neurological level, injury mechanism, intervention timing, surgical management, mean arterial pressure (MAP) management, outcome instruments, and follow-up were inconsistently and incompletely reported across studies. Mechanism of injury could not be assessed, many planned restriction analyses were infeasible or retained an identical network, and transitivity remains uncertain. Fourth, substantial between-study heterogeneity was detected (τ = 1.11 for overall AIS improvement and 8.65 points for overall motor score), and only four of 12 networks contained loops, limiting the statistical power of inconsistency diagnostics. Fifth, formal CINeMA certainty grading was not feasible because of the absence of comparison-specific contribution matrices, contribution-weighted risk-of-bias judgments, and prespecified clinical equivalence ranges; the structured domain appraisal in Supplementary Appendix 8 does not constitute a formal evidence-certainty grade. Finally, MAP management protocols were explicitly reported in only seven of 20 studies, so balanced co-intervention delivery across study arms and cohorts could not be assumed. Current clinical guidelines suggest augmenting MAP to at least 75–80 mmHg, but not actively beyond 90–95 mmHg, for 3–7 days; these are weak recommendations based on very-low-certainty evidence (Kwon et al., 2024). Collectively, these limitations preclude firm conclusions regarding comparative effectiveness and safety across evaluated neuroprotective regimens.

The present findings align with and contextualize recent evidence syntheses in ASCI neuroprotection. Serag et al. performed a broad systematic review of diverse neuroprotective agents, whereas Migliorini et al. focused on pharmacological and anti-inflammatory strategies targeting secondary SCI pathophysiology. These prior reviews consistently emphasized heterogeneity in injury phenotypes, therapeutic intervention windows, and outcome definitions, alongside low overall clinical certainty. In contrast, the present study used a Bayesian network meta-analysis to compare four prespecified immunomodulatory agents using comparative clinical data. The 2026 narrative review was published after the prespecified literature search cutoff and was therefore incorporated only for contextual discussion, rather than as primary evidence (Serag et al., 2024; Migliorini et al., 2025; Migliorini et al., 2026). Previous systematic reviews have also separately synthesized the evidence for riluzole and G-CSF (Weisbrod et al., 2024a; Weisbrod et al., 2024b).

The RISCIS trial randomized 193 participants with acute cervical traumatic SCI to riluzole or placebo but terminated early prior to full planned enrollment and failed to meet its primary 180-day upper-extremity motor endpoint, with no identifiable drug-related serious adverse events (Fehlings et al., 2023). Riluzole was not included as a node in the present analysis because it was outside the intervention domain applied in this review. Post hoc incorporation of an additional agent would have required *de novo* literature searching, screening, data extraction, and network model reconstruction. This scope decision should not be interpreted as evidence of riluzole ineffectiveness.

The principal strength of this review is its standardized, quantitative cross-agent comparative framework, which transparently reports relative estimates, posterior uncertainty, heterogeneity, predictive intervals, and model diagnostics for both neurological and safety outcomes. The curated study-level table distinguishes randomized, concurrent, historical, and sequential comparative designs while preserving original outcomes terminology from primary reports. These methodological enhancements improve analytical transparency but do not overcome the inherent limitations of a sparse, clinically heterogeneous evidence base.

## Conclusion

This Bayesian network meta-analysis provides comparative effectiveness and safety estimates for five regimens based on four immunomodulatory agents for acute SCI. G-CSF demonstrated a preliminary signal for neurological grade improvement. However, wide statistical uncertainty, risk of bias, nonconcurrent controls, potential participant cohort overlap, substantial between-study heterogeneity, and unresolved transitivity preclude a conclusion of definitive clinical superiority for G-CSF. All treatment rankings represent exploratory model-derived summaries and must be interpreted with extreme caution. Future trials should adopt adequately powered randomized controlled designs, implement prespecified stratification by baseline AIS grade and neurological injury level, standardized reporting of intervention timing, surgical interventions, and MAP management protocols, and employ harmonized neurological and patient-centered outcomes, alongside complete safety surveillance. Direct head-to-head evaluation of combination regimens is also warranted. Notably, the present evidence base lacked data on G-CSF combined with minocycline, and therapeutic synergy between these agents cannot be assumed in clinical practice.

## Data Availability

The aggregate analysis dataset generated from published reports during this study is not publicly available but can be obtained from the corresponding author on reasonable request. All source studies are cited in the article.
