# Supplementary material for: Comparative efficacy and safety of four immunomodulatory agents for acute spinal cord injury: a systematic review and Bayesian network meta-analysis
Source: Front Pharmacol. 2026 Oct 1;17:1942918. doi: 10.3389/fphar.2026.1942918 (PMC13668820; doi:10.3389/fphar.2026.1942918)
Supplement: Supplementary file 1 [file DataSheet1.pdf]

Appendix 1. Search Terms

MEDLINE (Pubmed)

MP

#1 (“spinal cord” OR spinal-cord* OR spine OR spinal) AND (Broken or break* OR fractur* OR wound* OR trauma* OR injur* OR damag* OR lesion* OR contusion* OR laceration* OR trauma OR ischemi*)

#2 paraplegi* OR paraparesis OR qadriplegi* OR quadriparesi* OR tetraplegi* OR tetraplagi* OR tetraparesis

#3 central cord injury syndrome

#4 (spine OR spinal) AND (myelopathy AND (traumatic OR post-traumatic))

#5 #1 OR #2 OR #3 OR #4

#6("Methylprednisolone"[Mesh]) OR ((6-Methylprednisolone[Title/Abstract]) OR (6 Methylprednisolone[Title/Abstract]) OR (Metipred[Title/Abstract]) OR (Medrol[Title/Abstract]) OR (Urbason[Title/Abstract]))

#7 #5 AND #6

#8 clinical trial[Publication Type] OR clinical trial[Title/Abstract] OR clinical trial[MeSH Terms] OR randomized controlled trial[Publication Type] OR randomized[Title/Abstract] OR placebo[Title/Abstract]

#9 #7 AND #8

EPO

#1 (“spinal cord” OR spinal-cord* OR spine OR spinal) AND (Broken or break* OR fractur* OR wound* OR trauma* OR injur* OR damag* OR lesion* OR contusion* OR laceration* OR trauma OR ischemi*)

#2 paraplegi* OR paraparesis OR qadriplegi* OR quadriparesi* OR tetraplegi* OR tetraplagi* OR tetraparesis

#3 central cord injury syndrome

#4 (spine OR spinal) AND (myelopathy AND (traumatic OR post-traumatic))

#5 #1 OR #2 OR #3 OR #4

#6("Erythropoietin"[Mesh]) OR ((EPO[Title/Abstract]) OR (Erythropoietin[Title/Abstract]) OR (recombinant human erythropoietin[Title/Abstract]) OR (rhEPO[Title/Abstract]))

#7 #5 AND #6

#8 clinical trial[Publication Type] OR clinical trial[Title/Abstract] OR clinical trial[MeSH Terms] OR randomized controlled trial[Publication Type] OR randomized[Title/Abstract] OR placebo[Title/Abstract]

#9 #7 AND #8

G-CSF

#1 (“spinal cord” OR spinal-cord* OR spine OR spinal) AND (Broken or break* OR fractur* OR wound* OR trauma* OR injur* OR damag* OR lesion* OR contusion* OR laceration* OR trauma OR ischemi*)

#2 paraplegi* OR paraparesis OR qadriplegi* OR quadriparesi* OR tetraplegi* OR tetraplagi* OR tetraparesis

#3 central cord injury syndrome

#4 (spine OR spinal) AND (myelopathy AND (traumatic OR post-traumatic))

#5 #1 OR #2 OR #3 OR #4

#6("Colony-Stimulating Factors"[Mesh]) OR ((Colony Stimulating Factors[Title/Abstract]) OR (Colony Stimulating Factor[Title/Abstract]) OR (Colony-Stimulating Factor[Title/Abstract]) OR (MGI-1 Protein[Title/Abstract]) OR (MGI 1 Protein[Title/Abstract]) OR (MGI-1[Title/Abstract]) OR (Myeloid Cell-Growth Inducer[Title/Abstract]) OR (Cell-Growth Inducer, Myeloid[Title/Abstract]) OR (Inducer, Myeloid Cell-Growth[Title/Abstract]) OR (Myeloid Cell Growth Inducer[Title/Abstract]) OR (Macrophage-Granulocyte Inducer[Title/Abstract]) OR (Inducer, Macrophage-Granulocyte[Title/Abstract]) OR (Macrophage Granulocyte Inducer[Title/Abstract]) OR (Protein Inducer MGI[Title/Abstract]) OR (MGI, Protein Inducer[Title/Abstract]))

#7 #5 AND #6

#8 clinical trial[Publication Type] OR clinical trial[Title/Abstract] OR clinical trial[MeSH Terms] OR randomized controlled trial[Publication Type] OR randomized[Title/Abstract] OR placebo[Title/Abstract]

#9 #7 AND #8

Minocycline

#1 (“spinal cord” OR spinal-cord* OR spine OR spinal) AND

(Broken OR break* OR fractur* OR wound* OR trauma* OR injur* OR

damag* OR lesion* OR contusion* OR laceration* OR trauma OR ischemi*)

#2 paraplegi* OR paraparesis OR qadriplegi* OR quadriparesi* OR

tetraplegi* OR tetraplagi* OR tetraparesis

#3 central cord injury syndrome

#4 (spine OR spinal) AND (myelopathy AND (traumatic OR post-traumatic))

#5 #1 OR #2 OR #3 OR #4

#6 ("Minocycline"[Mesh]) OR

((Minocycline[Title/Abstract]) OR

(Minocin[Title/Abstract]) OR

(Minomycin[Title/Abstract]) OR

(Minocycline Hydrochloride[Title/Abstract]))

#7 #5 AND #6

#8 clinical trial[Publication Type] OR

clinical trial[Title/Abstract] OR

clinical trial[MeSH Terms] OR

randomized controlled trial[Publication Type] OR

randomized[Title/Abstract] OR

placebo[Title/Abstract]

#9 #7 AND #8

EMBASE (Ovid)

MP

1.’spinal cord’/exp

2. 'spinal cord injury'/exp

3. 'spinal cord ischemia'/exp

4. 'central cord syndrome'/exp

5. (myelopathy NEAR/3 (traumatic OR post-traumatic)):ab,ti

6. ((spine OR spinal) NEAR/3 (fracture* OR wound* OR trauma* OR injur* OR damag*)):ab,ti

7. (spinal cord NEAR/3 (contusion OR laceration OR transaction OR trauma OR ischemia)):ab,ti

8. 'central cord injury syndrome':ab,ti

9. 'central spinal cord syndrome':ab,ti

10. 'paraplegia'/exp

11. 'quadriplegia'/exp

12. (paraplegi* OR quadriplegi* OR tetraplegi*):ab,ti

13. SCI:ab,ti

14. #1 OR #2 OR #3 OR #4 OR #5 OR #6 OR #7 OR #8 OR #9 OR #10 OR #11 OR #12 OR #13

15. 'methylprednisolone'/exp

16. (11,17 dihydroxy 17 (2 hydroxyacetyl) 6,10,13 trimethyl 7,8,9,11,12,14,15,16 octahydro 6h cyclopenta phenanthren 3 one* OR

11,17,21 trihydroxy 6 methylpregna 1,4 diene 3,20 dione* OR

11beta,17alpha,21 trihydroxy 6alpha methyl 1,4 pregnadiene 3,20 dione* OR

2 methylprednisolone* OR 5 methylprednisolone* OR

6alpha methylprednisolone* OR 6 methyl delta 1 hydrocortisone* OR

6 methyl prednisolone* OR 6 methylprednisolone* OR

6alpha methyl 11beta,17alpha,21 triol 1,4 pregnadiene 3,20 dione* OR

6alpha methyl delta1 hydrocortisone* OR 6alpha methylprednisolone* OR

adlone-40* OR adlone-80* OR beta methylprednisolone* OR

dep medalone 80* OR depmedalone* OR depoject-80* OR

depopred* OR esametone* OR firmacort* OR med-jec-40* OR

medixon* OR mednin* OR medralone 80* OR medrate* OR

medrol* OR medrol a* OR medrol adt pak* OR

medrol compositum* OR medrol dosepak* OR

medrol medules* OR medrol pak* OR medrone* OR

meprednisolone* OR meprelon* OR mesopren* OR

methacort 40* OR methacort 80* OR methyl prednisolone* OR

methylcotol* OR methylcotolone* OR methylpred dp* OR

methylsterolone* OR metidrol* OR metrisone* OR

metycortin* OR metypred* OR metypresol* OR

neomedrone* OR nsc 19987* OR nsc19987* OR

prednol* OR solomet* OR solu decortin* OR urbason* OR

methylprednisolone*):ab,ti

17. #15 OR #16

18. #14 AND #17

19. 'randomized controlled trial'/exp

20. 'controlled clinical trial'/exp

21. randomi?ed:ab,ti

22. placebo:ab

23. 'clinical trial'/exp

24. randomly:ab

25. trial:ti

26. #19 OR #20 OR #21 OR #22 OR #23 OR #24 OR #25

27. 'animal'/exp NOT ('human'/exp AND 'animal'/exp)

28. #26 NOT #27

29. #18 AND #28

EPO

1.’spinal cord’/exp

2. 'spinal cord injury'/exp

3. 'spinal cord ischemia'/exp

4. 'central cord syndrome'/exp

5. (myelopathy NEAR/3 (traumatic OR post-traumatic)):ab,ti

6. ((spine OR spinal) NEAR/3 (fracture* OR wound* OR trauma* OR injur* OR damag*)):ab,ti

7. (spinal cord NEAR/3 (contusion OR laceration OR transaction OR trauma OR ischemia)):ab,ti

8. 'central cord injury syndrome':ab,ti

9. 'central spinal cord syndrome':ab,ti

10. 'paraplegia'/exp

11. 'quadriplegia'/exp

12. (paraplegi* OR quadriplegi* OR tetraplegi*):ab,ti

13. SCI:ab,ti

14. #1 OR #2 OR #3 OR #4 OR #5 OR #6 OR #7 OR #8 OR #9 OR #10 OR #11 OR #12 OR #13

15. 'erythropoietin'/exp

16. (erthropoietin* OR erythropoiesis stimulating factor* OR erythropoietic factor* OR erythropoietic stimulation factor* OR hematopoietin* OR hemopoietin* OR kidney erythropoietic factor* OR renal erythropoietic factor* OR erythropoietin*):ab,ti

17. #15 OR #16

18. #14 AND #17

19. 'randomized controlled trial'/exp

20. 'controlled clinical trial'/exp

21. randomi?ed:ab,ti

22. placebo:ab

23. 'clinical trial'/exp

24. randomly:ab

25. trial:ti

26. #19 OR #20 OR #21 OR #22 OR #23 OR #24 OR #25

27. 'animal'/exp NOT ('human'/exp AND 'animal'/exp)

28. #26 NOT #27

29. #18 AND #28

G-CSF

1.'spinal cord'/exp

2. 'spinal cord injury'/exp

3. 'spinal cord ischemia'/exp

4. 'central cord syndrome'/exp

5. (myelopathy NEAR/3 (traumatic OR post-traumatic)):ab,ti

6. ((spine OR spinal) NEAR/3 (fracture* OR wound* OR trauma* OR injur* OR damag*)):ab,ti

7. (spinal cord NEAR/3 (contusion OR laceration OR transaction OR trauma OR ischemia)):ab,ti

8. 'central cord injury syndrome':ab,ti

9. 'central spinal cord syndrome':ab,ti

10. 'paraplegia'/exp

11. 'quadriplegia'/exp

12. (paraplegi* OR quadriplegi* OR tetraplegi*):ab,ti

13. SCI:ab,ti

14. #1 OR #2 OR #3 OR #4 OR #5 OR #6 OR #7 OR #8 OR #9 OR #10 OR #11 OR #12 OR #13

15. 'colony stimulating factor'/exp

16. (colony stimulating activity* OR colony stimulating factors* OR colony-stimulating factors* OR colony stimulating factor*):ab,ti

17. #15 OR #16

18. #14 AND #17

19. 'randomized controlled trial'/exp

20. 'controlled clinical trial'/exp

21. randomi?ed:ab,ti

22. placebo:ab

23. 'clinical trial'/exp

24. randomly:ab

25. trial:ti

26. #19 OR #20 OR #21 OR #22 OR #23 OR #24 OR #25

27. 'animal'/exp NOT ('human'/exp AND 'animal'/exp)

28. #26 NOT #27

29. #18 AND #28

Minocycline

1. 'spinal cord'/exp

2. 'spinal cord injury'/exp

3. 'spinal cord ischemia'/exp

4. 'central cord syndrome'/exp

5. (myelopathy NEAR/3 (traumatic OR post-traumatic)):ab,ti

6. ((spine OR spinal) NEAR/3 (fracture* OR wound* OR trauma* OR injur* OR damag*)):ab,ti

7. (spinal cord NEAR/3 (contusion OR laceration OR transaction OR trauma OR ischemia)):ab,ti

8. 'central cord injury syndrome':ab,ti

9. 'central spinal cord syndrome':ab,ti

10. 'paraplegia'/exp

11. 'quadriplegia'/exp

12. (paraplegi* OR quadriplegi* OR tetraplegi*):ab,ti

13. SCI:ab,ti

14. #1 OR #2 OR #3 OR #4 OR #5 OR #6 OR #7 OR #8 OR #9 OR #10 OR #11 OR #12 OR #13

15. 'minocycline'/exp

16. (minocycline* OR

minocin* OR

minomycin* OR

minocycline hydrochloride*):ab,ti

17. #15 OR #16

18. #14 AND #17

19. 'randomized controlled trial'/exp

20. 'controlled clinical trial'/exp

21. randomi?ed:ab,ti

22. placebo:ab

23. 'clinical trial'/exp

24. randomly:ab

25. trial:ti

26. #19 OR #20 OR #21 OR #22 OR #23 OR #24 OR #25

27. 'animal'/exp NOT ('human'/exp AND 'animal'/exp)

28. #26 NOT #27

29. #18 AND #28

Cochrane Central Register of Controlled Trials (CENTRAL) in The Cochrane Library (Wiley)

MP

#1 paraplegi* or paraparesis

#2 qadriplegi* or quadriparesi*

#3 tetraplegi* or tetraplagi* or tetraparesis

#4 (spine or spinal) near3 (Broken or break* or fracture* or wound* or trauma* or injur* or damag*)

#5 (spinal cord) near3 (contusion or laceration or trauma or injur* or ischemi*)

#6 (central cord injury syndrome)

#7 (myelopathy near3 (traumatic or post-traumatic))

#8 MeSH descriptor Central Cord Syndrome explode all trees

#9 MeSH descriptor Spinal Cord Ischemia explode all trees

#10 MeSH descriptor Spinal Fractures explode all trees

#11 MeSH descriptor Spinal Cord Injuries explode all trees

#12 MeSH descriptor Paraplegia explode all trees

#13 MeSH descriptor Quadriplegia explode all trees

#14 MeSH descriptor Spinal Cord explode all trees

#15 MeSH descriptor Cervical Vertebrae explode all trees

#16 (#1 OR #2 OR #3 OR #4 OR #5 OR #6 OR #7 OR #7 OR #8 OR #9 OR #10 OR #11 OR #12 OR #13 OR #14 OR #15)

#17 MeSH descriptor Methylprednisolone explode all trees

#18 Urbason* or Medrol* or 6-Methylprednisolone* or 6 Methylprednisolone* or Metipred*

#19 (#17 OR #18)

#20 (#16 AND #19)

EPO

#1 paraplegi* or paraparesis

#2 qadriplegi* or quadriparesi*

#3 tetraplegi* or tetraplagi* or tetraparesis

#4 (spine or spinal) near3 (Broken or break* or fracture* or wound* or trauma* or injur* or damag*)

#5 (spinal cord) near3 (contusion or laceration or trauma or injur* or ischemi*)

#6 (central cord injury syndrome)

#7 (myelopathy near3 (traumatic or post-traumatic))

#8 MeSH descriptor Central Cord Syndrome explode all trees

#9 MeSH descriptor Spinal Cord Ischemia explode all trees

#10 MeSH descriptor Spinal Fractures explode all trees

#11 MeSH descriptor Spinal Cord Injuries explode all trees

#12 MeSH descriptor Paraplegia explode all trees

#13 MeSH descriptor Quadriplegia explode all trees

#14 MeSH descriptor Spinal Cord explode all trees

#15 MeSH descriptor Cervical Vertebrae explode all trees

#16 (#1 OR #2 OR #3 OR #4 OR #5 OR #6 OR #7 OR #7 OR #8 OR #9 OR #10 OR #11 OR #12 OR #13 OR #14 OR #15)

#17 MeSH descriptor Erythropoietin explode all trees

#18 Recombinant 1 165 Erythropoietin, Glycoform Alpha* or Hexal, Epoetin Alfa* or Epoetin Alfa Hexal* or Alfa Hexal, Epoetin* or Alfa, Epoetin* or HX575 Compound* or Compound, HX575* or Epogen* or Eprex* or Binocrit* or Procrit* or HX575* or Heberitro* or EPO*

#19 (#17 OR #18)

#20 (#16 AND #19)

G-CSF

#1 paraplegi* or paraparesis

#2 qadriplegi* or quadriparesi*

#3 tetraplegi* or tetraplagi* or tetraparesis

#4 (spine or spinal) near3 (Broken or break* or fracture* or wound* or trauma* or injur* or damag*)

#5 (spinal cord) near3 (contusion or laceration or trauma or injur* or ischemi*)

#6 (central cord injury syndrome)

#7 (myelopathy near3 (traumatic or post-traumatic))

#8 MeSH descriptor Central Cord Syndrome explode all trees

#9 MeSH descriptor Spinal Cord Ischemia explode all trees

#10 MeSH descriptor Spinal Fractures explode all trees

#11 MeSH descriptor Spinal Cord Injuries explode all trees

#12 MeSH descriptor Paraplegia explode all trees

#13 MeSH descriptor Quadriplegia explode all trees

#14 MeSH descriptor Spinal Cord explode all trees

#15 MeSH descriptor Cervical Vertebrae explode all trees

#16 (#1 OR #2 OR #3 OR #4 OR #5 OR #6 OR #7 OR #7 OR #8 OR #9 OR #10 OR #11 OR #12 OR #13 OR #14 OR #15)

#17 MeSH descriptor Granulocyte Colony-Stimulating Factor explode all trees

#18 Colony-Stimulating Factor, Granulocyte* or Factor, Granulocyte Colony-Stimulating* or Growth Factor, Myeloid* or Colony Stimulating Factor, Granulocyte* or Myeloid Growth Factor* or G-CSF* or Granulocyte Colony Stimulating Factor* or Factor, Myeloid Growth*

#19 (#17 OR #18)

#20 (#16 AND #19)

Minocycline

#1 paraplegi* or paraparesis

#2 qadriplegi* or quadriparesi*

#3 tetraplegi* or tetraplagi* or tetraparesis

#4 (spine or spinal) near3

(Broken or break* or fracture* or wound* or trauma* or injur* or damag*)

#5 (spinal cord) near3

(contusion or laceration or trauma or injur* or ischemi*)

#6 (central cord injury syndrome)

#7 (myelopathy near3 (traumatic or post-traumatic))

#8 MeSH descriptor Central Cord Syndrome explode all trees

#9 MeSH descriptor Spinal Cord Ischemia explode all trees

#10 MeSH descriptor Spinal Fractures explode all trees

#11 MeSH descriptor Spinal Cord Injuries explode all trees

#12 MeSH descriptor Paraplegia explode all trees

#13 MeSH descriptor Quadriplegia explode all trees

#14 MeSH descriptor Spinal Cord explode all trees

#15 MeSH descriptor Cervical Vertebrae explode all trees

#16 (#1 OR #2 OR #3 OR #4 OR #5 OR #6 OR #7 OR #8 OR #9 OR #10 OR #11 OR #12 OR #13 OR #14 OR #15)

#17 MeSH descriptor Minocycline explode all tree

#18 Minocycline* OR

Minocin* OR

Minomycin* OR

Minocycline Hydrochloride*

#19 (#17 OR #18)

#20 (#16 AND #19)
