# Supplementary material for: Comparative efficacy and safety of four immunomodulatory agents for acute spinal cord injury: a systematic review and Bayesian network meta-analysis
Source: Front Pharmacol. 2026 Oct 1;17:1942918. doi: 10.3389/fphar.2026.1942918 (PMC13668820; doi:10.3389/fphar.2026.1942918)
Supplement: Supplementary file 2 [file DataSheet2.pdf]

Appendix 2. Risk of bias assessment for the individual studies.

A, RoB 2 risk of bias in individual randomized trials. B, RoB 2 risk of bias across randomized trials. C, ROBINS-I risk of bias in individual non-randomized studies. D, ROBINS-I risk of bias across non-randomized studies.

A. RoB 2 risk of bias in individual randomized trials.

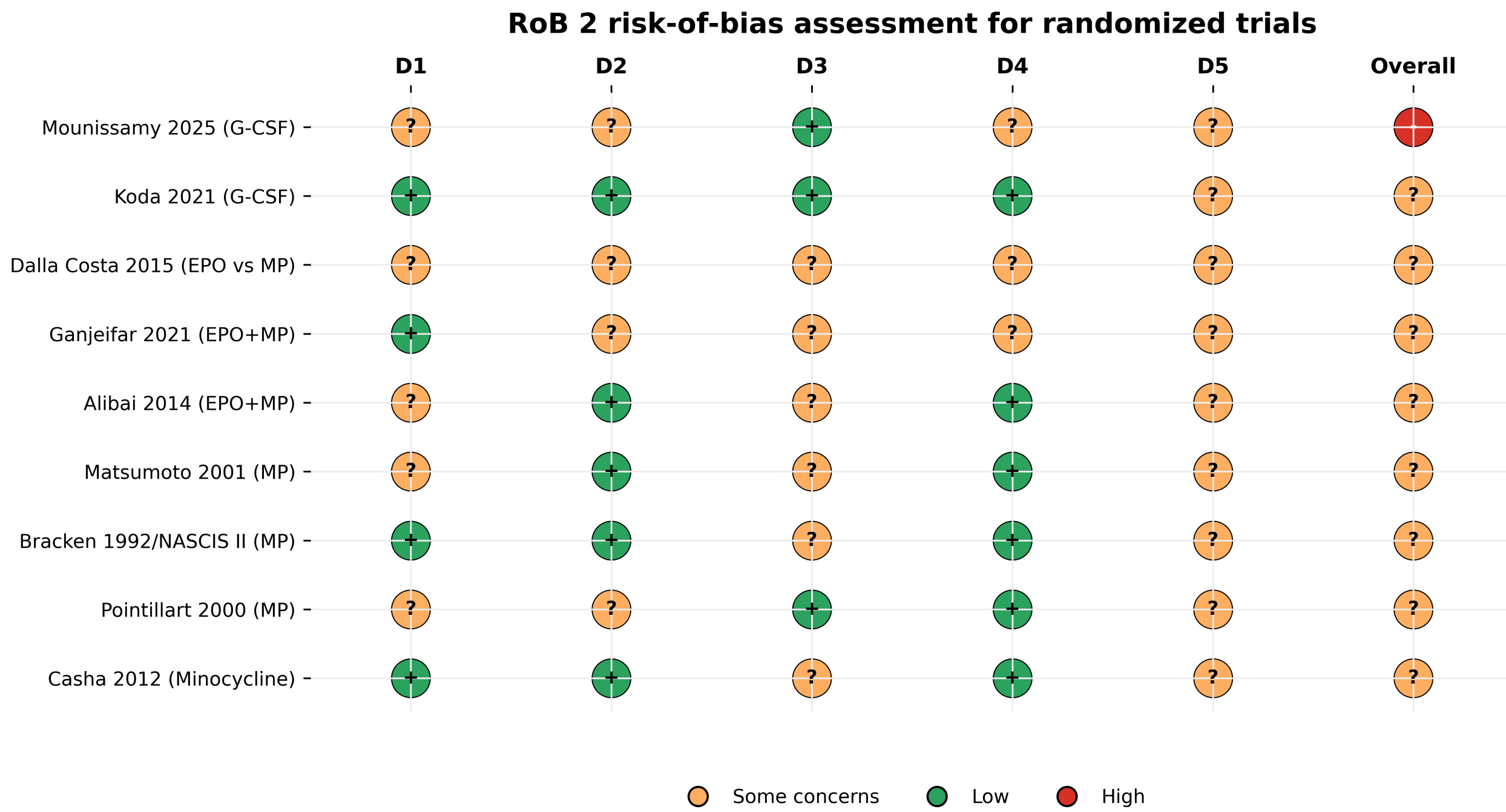

B. RoB 2 risk of bias across randomized trials.

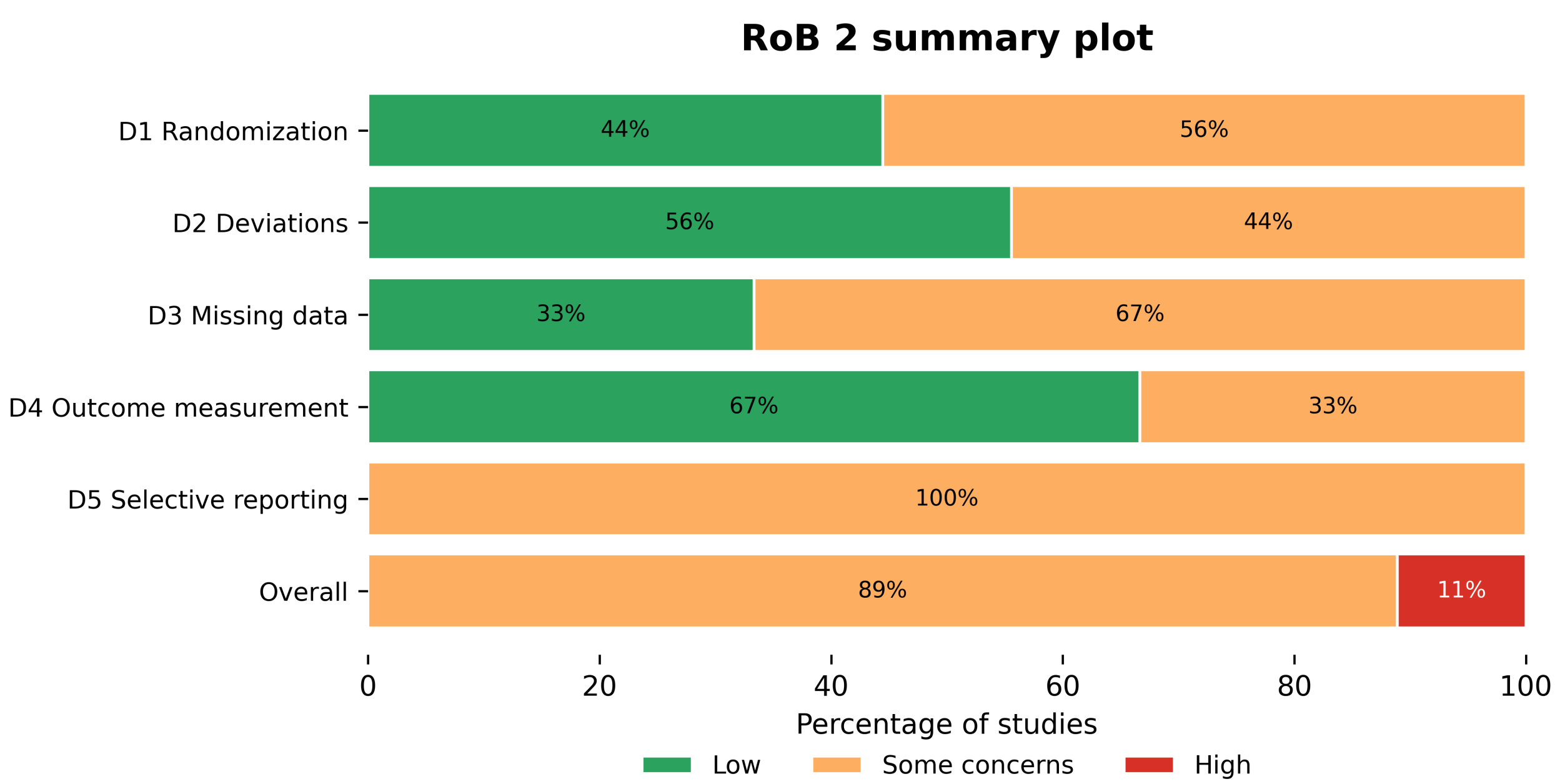

C. ROBINS-I risk of bias in individual non-randomized studies.

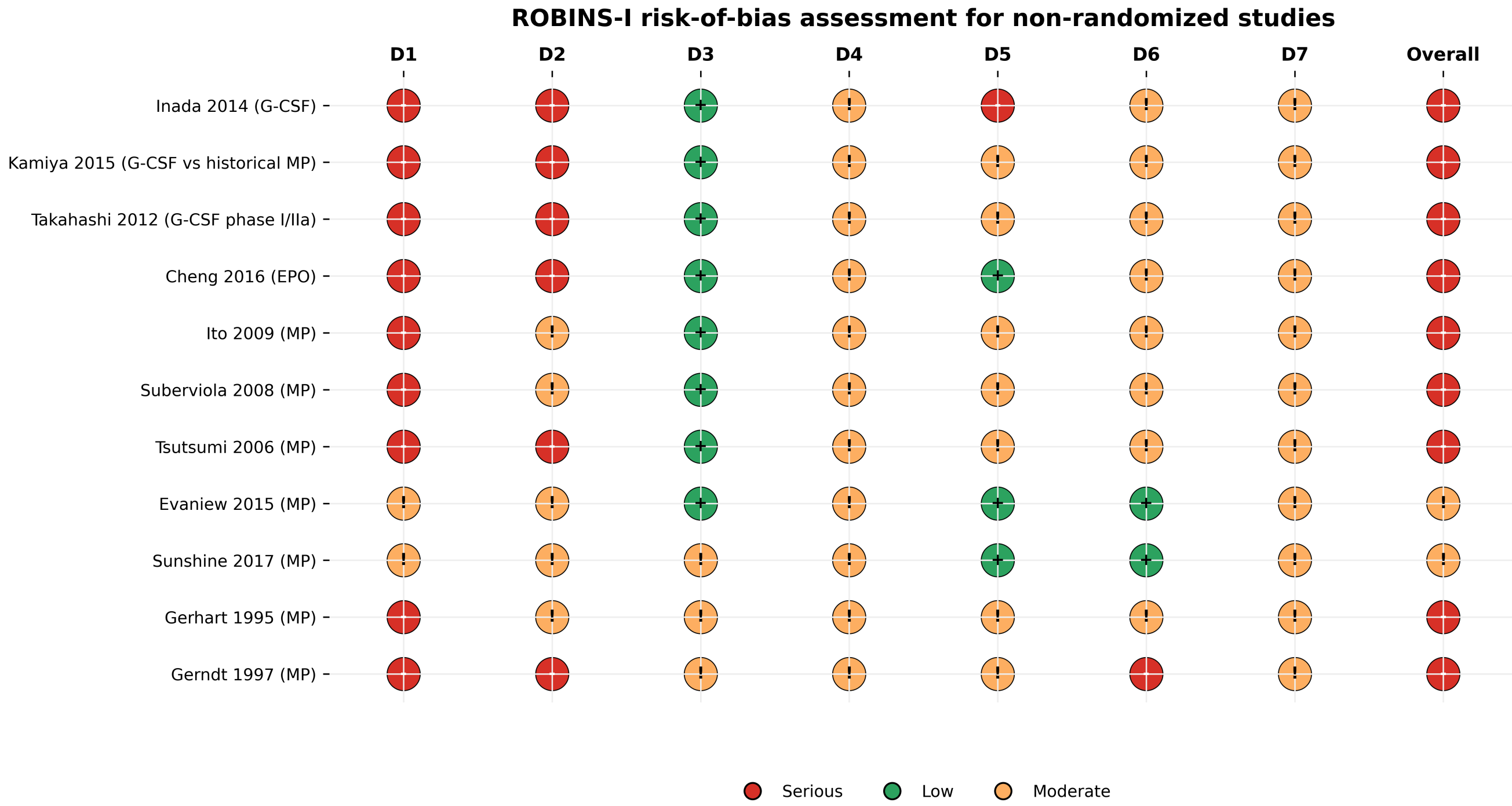

D. ROBINS-I risk of bias across non-randomized studies.

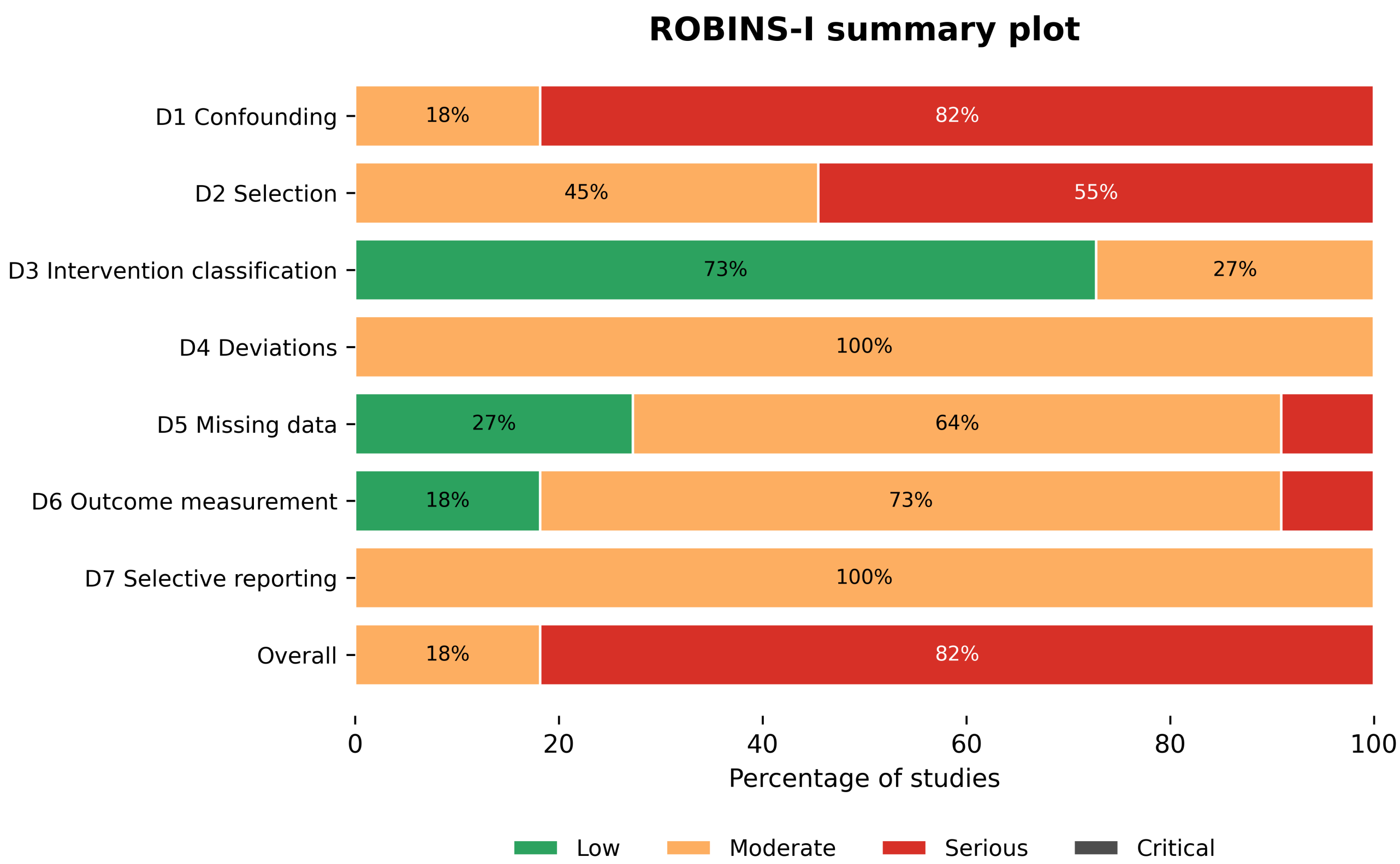

Summary of judgements.

Among the 9 randomized trials assessed using RoB 2, most domain-level judgements were low risk or had some concerns. The most frequent source of concern was incomplete reporting of allocation concealment, blinding, outcome reporting, or prespecified analysis plans. One randomized trial was judged as overall high risk because several domains, including randomization-related and reporting-related items, raised concerns. Among the 11 non-randomized studies assessed using ROBINS-I, the overall risk of bias was mainly serious, driven primarily by confounding, selection of participants, non-random treatment assignment, historical-control comparisons, and limited adjustment for baseline prognostic differences. Two adjusted registry or propensity-score-based studies were judged as overall moderate risk, but residual confounding could not be excluded.

Detailed risk-of-bias judgements for all included studies

Table S1. RoB 2 assessment for randomized trials.

Study	D1	D2	D3	D4	D5	Overall
Mounissamy 2025 (G-CSF)	Some concerns	Some concerns	Low	Some concerns	Some concerns	High
Koda 2021 (G-CSF)	Low	Low	Low	Low	Some concerns	Some concerns
Dalla Costa 2015 (EPO vs MP)	Some concerns	Some concerns	Some concerns	Some concerns	Some concerns	Some concerns
Ganjeifar 2021 (EPO+MP)	Low	Some concerns	Some concerns	Some concerns	Some concerns	Some concerns
Alibai 2014 (EPO+MP)	Some concerns	Low	Some concerns	Low	Some concerns	Some concerns
Matsumoto 2001 (MP)	Some concerns	Low	Some concerns	Low	Some concerns	Some concerns
Bracken 1992/NASCIS II (MP)	Low	Low	Some concerns	Low	Some concerns	Some concerns
Pointillart 2000 (MP)	Some concerns	Some concerns	Low	Low	Some concerns	Some concerns
Casha 2012 (Minocycline)	Low	Low	Some concerns	Low	Some concerns	Some concerns

RoB 2 domains: D1, bias arising from the randomization process; D2, bias due to deviations from intended interventions; D3, bias due to missing outcome data; D4, bias in measurement of the outcome; D5, bias in selection of the reported result.

Table S2. ROBINS-I assessment for non-randomized studies.

Study	D1	D2	D3	D4	D5	D6	D7	Overall
Inada 2014 (G-CSF)	Serious	Serious	Low	Moderate	Serious	Moderate	Moderate	Serious
Kamiya 2015 (G-CSF vs historical MP)	Serious	Serious	Low	Moderate	Moderate	Moderate	Moderate	Serious
Takahashi 2012 (G-CSF phase I/IIa)	Serious	Serious	Low	Moderate	Moderate	Moderate	Moderate	Serious
Cheng 2016 (EPO)	Serious	Serious	Low	Moderate	Low	Moderate	Moderate	Serious
Ito 2009 (MP)	Serious	Moderate	Low	Moderate	Moderate	Moderate	Moderate	Serious
Suberviola 2008 (MP)	Serious	Moderate	Low	Moderate	Moderate	Moderate	Moderate	Serious
Tsutsumi 2006 (MP)	Serious	Serious	Low	Moderate	Moderate	Moderate	Moderate	Serious
Evaniew 2015 (MP)	Moderate	Moderate	Low	Moderate	Low	Low	Moderate	Moderate
Sunshine 2017 (MP)	Moderate	Moderate	Moderate	Moderate	Low	Low	Moderate	Moderate
Gerhart 1995 (MP)	Serious	Moderate	Moderate	Moderate	Moderate	Moderate	Moderate	Serious
Gerndt 1997 (MP)	Serious	Serious	Moderate	Moderate	Moderate	Serious	Moderate	Serious

ROBINS-I domains: D1, bias due to confounding; D2, bias in selection of participants into the study; D3, bias in classification of interventions; D4, bias due to deviations from intended interventions; D5, bias due to missing data; D6, bias in measurement of outcomes; D7, bias in selection of the reported result.
