# Supplementary material for: Comparative efficacy and safety of four immunomodulatory agents for acute spinal cord injury: a systematic review and Bayesian network meta-analysis
Source: Front Pharmacol. 2026 Oct 1;17:1942918. doi: 10.3389/fphar.2026.1942918 (PMC13668820; doi:10.3389/fphar.2026.1942918)
Supplement: Supplementary file 3 [file DataSheet3.pdf]

Supplementary Appendix 3. Exploratory assessment of small-study effects and reporting bias

Comparison-adjusted funnel plots and regression-based asymmetry tests were examined for the four primary outcomes. These diagnostics are exploratory. A nonsignificant asymmetry test does not establish the absence of publication or selective-reporting bias, particularly when networks are sparse, study designs and effect sizes are heterogeneous, and potentially linked reports may not be statistically independent. Publication year was not consistently available, so time-lag analyses were not performed. The plotted count is the number of outcome-specific effect-size records, not a de-duplicated count of unique studies or participants; a report could contribute more than one point under the diagnostic data arrangement.

Outcome	Plotted reports/contrasts	Egger-type p value	Cautious interpretation
ASIA Motor Score	24	0.0001	Asymmetry signal present; compatible with small-study effects, selective reporting, heterogeneity, or design differences.
AIS grade improvement	22	0.1857	No statistical asymmetry signal detected; reporting bias remains uncertain.
Overall adverse events	12	0.7415	No statistical asymmetry signal detected; sparse and heterogeneous safety reporting limits inference.
Infection	14	0.9385	No statistical asymmetry signal detected; sparse outcome reporting limits inference.

Overall interpretation: the motor-score network showed a clear asymmetry signal, whereas the other tests did not. The latter findings should not be read as evidence that reporting bias is absent. Reporting bias was therefore retained as a concern in the structured qualitative certainty appraisal (Supplementary Appendix 8), and no treatment ranking was upgraded on the basis of these diagnostics.
