# Supplementary material for: Comparative efficacy and safety of four immunomodulatory agents for acute spinal cord injury: a systematic review and Bayesian network meta-analysis
Source: Front Pharmacol. 2026 Oct 1;17:1942918. doi: 10.3389/fphar.2026.1942918 (PMC13668820; doi:10.3389/fphar.2026.1942918)
Supplement: Supplementary file 4 [file DataSheet4.pdf]

Supplementary Appendix 4. Global and local inconsistency assessment

Inconsistency was evaluated only when the network contained a closed loop. Four of the 12 outcome-by-follow-up networks contained loops; the remaining eight were star-shaped or single comparisons and could not support inconsistency testing.

Global assessment

Network	Consistency − UME DIC	Interpretation
AIS overall	+1.046	Similar fit; AIS UME did not meet ESS criterion
AIS long-term	+0.860	Similar fit; AIS UME did not meet ESS criterion
ASIA Motor Score overall	−2.147	Similar fit
ASIA Motor Score long-term	−1.933	Similar fit

Absolute DIC differences were 0.86–2.15. These comparisons showed no clear global model-fit signal of inconsistency; they do not prove consistency, and the two AIS UME models were interpreted cautiously because they did not meet the prespecified ESS criterion.

Local assessment

Node splitting was attempted for 20 splittable contrasts. Six were not interpreted: the EPO–methylprednisolone split in all four loop-containing networks because direct evidence came from a single extremely sparse or imprecise study, and the EPO–placebo split in the two AIS networks because its indirect estimate depended entirely on that unstable edge. Among the remaining 14 interpretable splits, all 95% CrIs for the inconsistency factor included zero and two-sided posterior tail-area probabilities ranged from 0.2317 to 0.9869.

The frequentist design-by-treatment between-design p values were 0.3010, 0.4369, 0.8788, and 0.3582 for the four loop-containing networks. Significant within-design Q components reflect heterogeneity, not inconsistency.

Full direct, indirect, network, inconsistency-factor, convergence, and frequentist cross-check results are provided in Supplementary Appendix 6, Tables S5–S9.
