# Supplementary material for: Comparative efficacy and safety of four immunomodulatory agents for acute spinal cord injury: a systematic review and Bayesian network meta-analysis
Source: Front Pharmacol. 2026 Oct 1;17:1942918. doi: 10.3389/fphar.2026.1942918 (PMC13668820; doi:10.3389/fphar.2026.1942918)
Supplement: Supplementary file 5 [file DataSheet5.pdf]

# Supplementary Appendix 5. Between-study heterogeneity and predictive uncertainty

Between-study heterogeneity was summarized as the posterior median between-study standard deviation τ with a 95% CrI. Values are scale-specific and must not be compared numerically across log-odds-ratio and point scales.

| Outcome | Follow-up | Scale | τ (95% CrI) |
|---|---|---|---|
| AIS grade improvement | Overall | Log odds ratio | 1.1102 (0.2398–2.7406) |
| AIS grade improvement | Short-term | Log odds ratio | 1.5669 (0.0801–3.0560) |
| AIS grade improvement | Long-term | Log odds ratio | 1.1365 (0.2556–2.3353) |
| ASIA Motor Score | Overall | Points | 8.6530 (3.7139–17.3603) |
| ASIA Motor Score | Short-term | Points | 9.2062 (3.2444–13.7537) |
| ASIA Motor Score | Long-term | Points | 9.7049 (4.1401–18.5481) |
| Overall adverse events | Overall | Log odds ratio | 0.5747 (0.0503–1.0391) |
| Overall adverse events | Short-term | Log odds ratio | 0.4278 (0.0231–0.8285) |
| Overall adverse events | Long-term | Log odds ratio | 0.6786 (0.0751–1.0554) |
| Infection | Overall | Log odds ratio | 0.8461 (0.2197–1.4660) |
| Infection | Short-term | Log odds ratio | 0.9567 (0.1896–1.4846) |
| Infection | Long-term | Log odds ratio | 0.4222 (0.0216–0.9546) |

Heterogeneity was substantial for the primary efficacy networks. Predictive intervals in Supplementary Appendix 6, Table S10 were consequently much wider than pooled-effect CrIs and frequently crossed the null. Random-effects estimates and rankings should therefore be interpreted as averages over heterogeneous studies, not effects expected uniformly in a future study.
