# Supplementary material for: Comparative efficacy and safety of four immunomodulatory agents for acute spinal cord injury: a systematic review and Bayesian network meta-analysis
Source: Front Pharmacol. 2026 Oct 1;17:1942918. doi: 10.3389/fphar.2026.1942918 (PMC13668820; doi:10.3389/fphar.2026.1942918)
Supplement: Supplementary file 6 [file DataSheet7.pdf]

Supplementary Appendix 7. Transitivity, effect-modifier reporting, and exploratory sensitivity analyses

Transitivity could not be verified formally. The table below summarizes what could be assessed from the source reports and the limitations of the available comparison-specific information. The subsequent restriction analyses are exploratory frequentist random-effects analyses; they are not substitutes for the primary Bayesian models and should not be interpreted as proving robustness.

Candidate effect modifier	Availability/handling	Implication for transitivity
Baseline AIS/Frankel severity	Inconsistently reported; mixed severity or incomplete-injury cohorts predominated. Reliable comparison-specific distributions were unavailable.	Potential imbalance could not be excluded; reliable network meta-regression was infeasible.
Neurological level	Cervical versus thoracic composition was incompletely reported and restrictions often removed a treatment node.	Residual concern; only limited exploratory restriction was feasible.
Mechanism of injury	Not captured consistently in the extracted comparative dataset.	Unassessable in this revision.
Intervention timing	Eligibility required acute SCI, but exact time from injury to treatment was variably reported.	Potential effect modification remains.
Surgical management	Decompression and operative approach were incompletely reported; across-arm balance could not be assumed when absent.	Potential co-intervention imbalance remains.
MAP management	Explicitly reported in 7/20 studies; no protocol was imputed for unreported studies.	Potential co-intervention imbalance remains.
Follow-up	One time point per study was selected within each analysis; connected short- and long-term networks were also analyzed.	Reduces duplicate contribution but does not remove between-study recovery-phase differences.
Outcome assessment	Prespecified neurological endpoints were analyzed separately; study terminology was retained and non-equivalent instruments were not pooled without a stated rule.	Measurement heterogeneity was reduced but not eliminated.

Exploratory fitted restriction scenarios

The following table reproduces the feasible exploratory frequentist restrictions. I^2 and τ are frequentist sensitivity-model quantities and must not be substituted for the Bayesian primary-model τ values in Supplementary Appendix 6.

Outcome (measure)	Follow-up	Scenario	k	Treat.	I^2 (%)	τ	Δ Dir.	Δ Sig.	Comment
AIS grade improvement (OR)	90 d	Primary network	6	4	59.5	0.67	ref	ref	Reference random-effects network meta-analysis.
		Baseline severity adequately characterised	5	4	0.0	0.00	1	0	Heterogeneity resolved; the single direction change (G-CSF vs Placebo) is in a comparison non-significant in the primary analysis and remaining so.
		Intervention timing adequately characterised	4	4	0.0	0.00	1	0	As above (G-CSF vs Placebo).
		Follow-up restricted to 90-day window	5	4	0.0	0.00	1	0	As above (G-CSF vs Placebo).

Outcome (measure)	Follow-up	Scenario	k	Treat.	I² (%)	τ	Δ Dir.	Δ Sig.	Comment
		Standardised outcome instrument	6	4	59.5	0.67	0	0	Identical to the primary analysis; no study removed.
ASIA motor score (MD)	90 d	Primary network	6	5	55.4	5.57	ref	ref	Reference; no treatment significant vs Placebo.
		Baseline severity adequately characterised	5	5	52.8	5.14	1	0	Direction change (EPO vs Placebo) among comparisons non-significant throughout.
		Intervention timing adequately characterised	5	5	57.7	6.01	1	0	Direction change (MP vs Placebo) among comparisons non-significant throughout.
		Follow-up restricted to 90-day window	6	5	55.4	5.57	0	0	Identical to the primary analysis; no study removed.
		Standardised outcome instrument	6	5	55.4	5.57	0	0	Identical to the primary analysis; no study removed.
ASIA motor score (MD)	180 d	Primary network	4	4	28.0	3.48	ref	ref	Reference. MP (MD 20.30, 95% CI 8.77 to 31.83) and G-CSF (MD 9.09, 95% CI 0.02 to 18.16) significant vs Placebo.
		Cervical injuries only	4	4	28.0	3.48	0	0	Restriction removed no study; estimates and heterogeneity identical to the primary analysis.
		Intervention timing adequately characterised	4	4	28.0	3.48	0	0	Identical to the primary analysis (no study removed).
		Follow-up restricted to 180-day window	4	4	28.0	3.48	0	0	Identical to the primary analysis (no study removed).
		Standardised outcome instrument	4	4	28.0	3.48	0	0	Identical to the primary analysis (no study removed).
ASIA motor score (MD)	365 d	Primary network	6	5	77.8	8.59	ref	ref	Reference; substantial heterogeneity.
		Baseline severity adequately characterised	4	5	n/e	n/e	0	1	Network saturated (0 residual df); τ and I² not estimable and the interval collapses to fixed-effect width. The apparent gain in significance for EPO vs Placebo is an artefact of this, not a treatment signal (point estimate unchanged at 6.80).

Outcome (measure)	Follow-up	Scenario	k	Treat.	I² (%)	τ	Δ Dir.	Δ Sig.	Comment
		Surgical management balanced across arms	5	5	86.5	10.00	0	0	Heterogeneity remained high.
		Follow-up restricted to 365-day window	6	5	77.8	8.59	0	0	Identical to the primary analysis (no study removed).
		Standardised outcome instrument	5	5	37.6	4.43	0	0	Heterogeneity reduced; no comparison changed direction or significance.

Direction changes were confined to comparisons that were nonsignificant before and after restriction. The single apparent significance change occurred in a saturated network with zero residual degrees of freedom and was not interpreted as a treatment signal. Several restrictions removed no study and therefore yielded an identical network by construction.

Prespecified restrictions that were infeasible

Outcome (measure)	Follow-up	Scenario	Feasibility status and reason
AIS grade improvement (OR)	90 d	High risk-of-bias studies excluded (Appendix 2)	Not estimable: too few connected studies remain after exclusion.
		Cervical injuries only	Not estimable: a treatment node (EPO) is lost.
		Surgical management balanced across arms	Not estimable: a treatment node is lost.
ASIA motor score (MD)	90 d	High risk-of-bias studies excluded (Appendix 2)	Not estimable: the evidence network becomes disconnected.
		Cervical injuries only	Not estimable: a treatment node (EPO) is lost.
		Surgical management balanced across arms	Not estimable: a treatment node is lost.
ASIA motor score (MD)	180 d	High risk-of-bias studies excluded (Appendix 2)	Not estimable: fewer than three studies remain.
		Baseline severity adequately characterised	Not estimable: fewer than three studies remain.
		Surgical management balanced across arms	Not estimable: fewer than three studies remain.
ASIA motor score (MD)	365 d	High risk-of-bias studies excluded (Appendix 2)	Not estimable: a treatment node is lost.
		Cervical injuries only	Not estimable: a treatment node (EPO) is lost.
		Intervention timing adequately characterised	Not estimable: a treatment node is lost.

Exclusion of high/serious risk-of-bias studies could not preserve any primary network. This indicates that higher-risk studies are pivotal connectors; it does not demonstrate that the full-network estimates are unbiased. Taken together, the descriptive and exploratory analyses support cautious interpretation but do not establish the transitivity assumption.
