# Supplementary material for: Comparative efficacy and safety of four immunomodulatory agents for acute spinal cord injury: a systematic review and Bayesian network meta-analysis
Source: Front Pharmacol. 2026 Oct 1;17:1942918. doi: 10.3389/fphar.2026.1942918 (PMC13668820; doi:10.3389/fphar.2026.1942918)
Supplement: Supplementary file 7 [file DataSheet8.pdf]

Supplementary Appendix 8. Structured qualitative certainty appraisal guided by CINeMA domains

This appendix is a structured qualitative appraisal guided by the six CINeMA domains: within-study bias, reporting bias, indirectness, imprecision, heterogeneity, and incoherence. It is not a formal CINeMA assessment or a GRADE certainty rating. Formal comparison-level grading requires a contribution matrix, contribution-weighted study-level risk-of-bias judgments, and prespecified clinically important or equivalence ranges; those inputs were not available for these sparse networks.

Outcome	Within-study bias	Reporting bias	Indirectness/transitivity	Imprecision	Heterogeneity	Incoherence	Overall qualitative interpretation
AIS grade improvement	Major concern: mixed randomized and nonrandomized evidence; serious ROBINS-I concerns; nonconcurrent controls.	Some/major concern: sparse comparisons and limited ability to assess selective nonpublication.	Major concern: key effect modifiers incompletely reported; transitivity uncertain.	Major concern: very wide CrIs; only G-CSF excluded the null and remained imprecise.	Major concern: τ 1.11 (0.24–2.74) on log-OR scale; wide predictive intervals.	Some concern: no clear signal among 14 interpretable splits, but six not interpretable and AIS UME ESS criterion not met.	Very limited confidence; exploratory signal only.
ASIA Motor Score	Major concern: mixed designs and serious residual confounding in nonrandomized studies.	Some/major concern: sparse networks and funnel asymmetry may reflect bias or heterogeneity.	Major concern: severity, level, timing, surgery, and assessment details incompletely balanced.	Major concern: every active-versus-control CrI included zero.	Major concern: τ 8.65 (3.71–17.36) points; wide predictive intervals.	Some concern: no clear signal in interpretable checks, but sparse loops and dependent tests limit power.	Very limited confidence; no established motor benefit.
Overall adverse events	Major concern: mixed designs, heterogeneous definitions, and sparse reporting.	Major concern: too few informative studies to assess reporting bias.	Major concern: outcome definitions and care pathways varied.	Major concern: G-CSF not reliably estimable; MP CrI crossed the null.	Some/major concern: τ 0.57 (0.05–1.04) on log-OR scale.	Not testable: the network had no closed loop.	Very limited confidence; no treatment ranking should be inferred.
Infection	Major concern: mixed designs and heterogeneous surveillance.	Major concern: too few informative studies to assess reporting bias.	Major concern: infection definitions and background care varied.	Major concern: all CrIs were wide and crossed the null.	Major concern: τ 0.85 (0.22–1.47) on log-OR scale.	Not testable: the network had no closed loop.	Very limited confidence; no clear safety difference.

Primary effect estimates considered in the appraisal

Outcome	Comparison versus placebo/standard care	Random-effects estimate (95% CrI)	Interpretation
AIS grade improvement	G-CSF	OR 5.12 (1.15–32.42)	Signal with substantial imprecision and bias/heterogeneity concerns
AIS grade improvement	EPO	OR 4.87 (0.44–135.90)	Very imprecise; includes null
AIS grade improvement	EPO + MP	OR 3.44 (0.09–164.02)	Very imprecise; includes null
AIS grade improvement	MP	OR 1.31 (0.30–5.72)	Imprecise; includes null
ASIA Motor Score	G-CSF	MD 11.49 (−0.44 to 23.49)	Imprecise; includes zero
ASIA Motor Score	Minocycline	MD 6.83 (−15.24 to 28.75)	Very imprecise; includes zero
ASIA Motor Score	EPO	MD 6.75 (−13.16 to 26.42)	Very imprecise; includes zero
ASIA Motor Score	MP	MD 2.28 (−6.24 to 10.94)	Imprecise; includes zero
Overall adverse events	G-CSF	Not reliably estimable	Near-complete-event study; posterior highly prior-sensitive
Overall adverse events	MP	OR 1.13 (0.58–2.34)	Imprecise; includes null
Infection	G-CSF	OR 0.93 (0.11–7.65)	Very imprecise; includes null
Infection	Minocycline	OR 0.38 (0.04–3.45)	Very imprecise; includes null
Infection	MP	OR 1.73 (0.69–4.77)	Imprecise; includes null
